# The general psychopathology factor (*p*) from adolescence to adulthood: Exploring the developmental trajectories of *p* using a multi-method approach

**DOI:** 10.1017/S0954579422000463

**Published:** 2022-07-11

**Authors:** Alexandria M. Choate, Marina A. Bornovalova, Alison E. Hipwell, Tammy Chung, Stephanie D. Stepp

**Affiliations:** 1 Department of Psychology, University of South Florida, Tampa, FL, USA; 2 Department of Psychiatry, University of Pittsburgh, Pittsburgh, PA, USA; 3 Department of Psychiatry, Institute for Health, Healthcare Policy and Aging Research; Rutgers, The State University of New Jersey, New Brunswick, NJ, USA

**Keywords:** adolescence, co-occurring psychopathology, *p*-factor, Pittsburgh Girls Study

## Abstract

Considerable attention has been directed towards studying co-occurring psychopathology through the lens of a general factor (*p*-factor). However, the developmental trajectory and stability of the *p*-factor have yet to be fully understood. The present study examined the explanatory power of dynamic mutualism theory – an alternative framework that suggests the *p*-factor is a product of lower-level symptom interactions that strengthen throughout development. Data were drawn from a population-based sample of girls (*N* = 2450) who reported on the severity of internalizing and externalizing problems each year from age 14 to age 21. Predictions of dynamic mutualism were tested using three distinct complementary statistical approaches including: longitudinal bifactor models, random-intercept cross-lagged panel models (RI-CLPMs), and network models. Across methods, study results document preliminary support for mutualistic processes in the development of co-occurring psychopathology (that is captured in *p*). Findings emphasize the importance of exploring alternative frameworks and methods for better understanding the *p*-factor and its development.

## Introduction

The prevalence of comorbid/co-occurring psychopathology has presented conceptual and methodological challenges for studying mental illness. Prior conceptualizations that classify mental disorders as discrete categories are undermined by the staggering rates of co-occurring^1^ psychopathology, with nearly 50% of those diagnosed with a mental disorder meeting criteria for at least one other disorder simultaneously (D. L. Newman et al., 1998). Because the co-variation of mental disorders has become the norm rather than the exception, the field has experienced a paradigm shift towards the use of broader transdiagnostic models, including a framework that theorizes disorders to stem from two latent vulnerabilities (i.e., internalizing and externalizing; Krueger et al., 1998).

The two-factor internalizing-externalizing structure has been extensively replicated, with these factors found to have moderate, positive correlations (e.g., M. D. Kramer et al., 2008; Krueger et al., 1998; Lahey et al., 2017). These observed correlations have in turn fostered researchers to search for a more global factor of psychopathology – termed “*p*” (Caspi et al., 2014) – that may account for the phenotypic stability and co-occurrence of various mental disorders (Caspi & Moffitt, 2018). Although a number of studies have found evidence for a general *p*-factor using different symptoms and age groups (e.g., Brandes et al., 2019; Gomez et al., 2019; Greene & Eaton, 2017; Laceulle et al., 2015; Lahey et al., 2012, 2015; Tackett et al., 2013; Watts et al., 2019), extensive debate has persisted surrounding the interpretability and utility of *p* (see Smith et al., 2020 for a review).

Similar to the general factor of intelligence (g-factor; Spearman, 1904), dominant conceptualizations of *p* have shown preference towards causal, or common cause positions that theorize *p* as a latent vulnerability which influences one’s propensity for developing psychopathology (Aristodemou & Fried, 2020; Caspi et al., 2014; Caspi & Moffitt, 2018; Levin-Aspenson et al., 2021; van Bork et al., 2017). Support for common cause interpretations of *p* have largely been based on replications of the *p*-factor, as well as evidence suggesting *p* is moderately heritable and predictive of several adverse clinical outcomes (e.g., Conway et al., 2019; Lahey et al., 2017; M. M. Martel et al., 2017; Pettersson et al., 2018).

Nonetheless, proponents of *p* as a causal or substantive entity have not gone without criticism, and several concerns have been raised with respect to the methodology and “weak” theories used to justify the validity of *p* (Bonifay et al., 2017; Fried et al., 2021; Fried, 2020; van Bork et al., 2017; Watts et al., 2020). For example, given that structural models are not a rigorous test of causality, nor are intended to “discover” the existence of latent constructs (Bollen & Lennox, 1991; Borsboom et al., 2003), a number of papers have highlighted issues with reifying the *p*-factor as a causal entity based on model fit or findings of a strong general factor (Watts et al., 2020; van Bork et al., 2017). However, these critiques are not necessarily unique to the *p*-factor and could apply to any latent variable model.

An additional concern related to the *p*-factor stems from the quantity of studies arguing that *p* reflects a substantive construct without ruling out alternative explanations. Stated differently, a large proportion of studies have centered their research questions around the assumption that *p* is a valid construct that explains the manifestation of psychopathology, rather than a variable in need of explanation (Fried et al., 2021). Yet, whether the statistical emergence of *p* is produced by an unobserved vulnerability or is attributable to an entirely different data generating process (e.g., result of local symptom interactions, communal impairment, etc.), has remained unclear. Given the research and clinical implications that an agreed upon interpretation of *p* may offer (Levin-Aspenson et al., 2021), it will be important for future work to adequately test and rule out alternative explanations behind the general *p*-factor.

Although there are other alternative explanations of *p* that go beyond the scope of this paper (e.g., Oltmanns et al., 2018; van Bork et al., 2017), one intriguing hypothesis first introduced in the intelligence literature suggests that *p* is a product of evolving symptom interactions, rather than the cause of them (Caspi et al., 2014). This theory, termed dynamic mutualism, suggests that the positive manifold underlying the *p*-factor may be caused by developing interactions between lower-level symptoms and other psychological, biological, and environmental processes (van der Maas et al., 2006). These processes are assumed to be independent in early childhood, though are predicted to form increasingly strong associations throughout development until a state of equilibrium is reached (van der Maas et al., 2006, 2017). Accordingly, the core predictions of mutualism theory challenge common cause positions of *p*, in addition to other developmental psychopathology theories. For example, the differentiation hypothesis directly opposes predictions of dynamic mutualism by stipulating that disorder co-variation should decrease with age as symptoms become more differentiated from one another over time (Lahey et al., 2004; Lilienfeld et al., 1994; Sterba et al., 2010). This hypothesis has been extended to the *p*-factor under the term “*p*-differentiation,” and is based on the assumption that *p* captures a general predisposition towards psychopathology that becomes increasingly specific with age (McElroy, Belsky, et al., 2018; Murray et al., 2016; Patalay et al., 2015).

To our knowledge, only two published studies have explicitly tested dynamic mutualism in the development of *p* by estimating variants of a bifactor model (McElroy, Belsky, et al., 2018; Murray et al., 2016). The bifactor model yields an indirect test of mutualism by evaluating whether the longitudinal strength and/or reliable variance accounted for by *p* increases, implying that the relations between lower-level symptoms are strengthening over time. Guided by this assumption, Murray et al. (2016) used an exploratory bifactor technique in a large, ethnically diverse sample of European children to extract a general *p*-factor and four specific factors based on teacher reports at eight time points (ages 7–15). Results were incongruent with mutualism, indicating that the strength of *p* and the specific factors were stable over time.

In a related study, dynamic mutualism theory was tested by estimating cross-sectional bifactor models across ages 2–14 using maternal self-reports on internalizing, externalizing, and attention-related symptoms. Results were in agreement with Murray et al. (2016), revealing high factor stability and a dominant *p*-factor that accounted for the most variance across development (McElroy, Belsky, et al. 2018). The authors also assessed the phenotypic stability of *p* and the specific factors by saving factor scores from the bifactor models to later use in a cross-lagged panel design. Findings from this model suggested that cross-lags were attenuated and less consistent compared to the autoregressive paths, and that *p* both predicted, and was predicted by, the specific factors at various ages (McElroy, Belsky, et al. 2018).

Despite the value of these studies, several limitations still preclude strong inferences on the plausibility of mutualism theory in the development of *p*. First, previous studies have relied on teacher and/or parent reported data, which are often misaligned with self-report data from children or adolescents (T. L. Kramer et al., 2004; Van Roy et al., 2010). Additionally, the analyzed age ranges in recent studies focused on earlier periods of development, with less research examining transitional periods from adolescence to adulthood, or young adulthood to middle-adulthood. However, conceptualizing psychopathology from a mutualism perspective implies that certain developmental windows or “sensitive periods” may be characterized by unique symptom dynamics, such that the strength of these interactions or types of interactions may differ across development (Kievit, 2020). This suggests that tailoring interventions to a specific developmental window may be an effective tactic for intervention (Forbes et al., 2019). Equally, research that can identify which developmental periods or transitions are most vulnerable to the expression and/or escalation of symptoms may further inform preventative approaches to psychopathology.

Second, mutualism theory makes intraindividual level predictions that are suggested to inform phenomena at the between- and within-person levels (van der Maas et al., 2006, 2017). Previous investigations that have solely used cross-sectional approaches (e.g., bifactor or cross-sectionally derived panel models) are thus ill-equipped to examine mutualistic processes due to the conflation of within and between-person effects. Multilevel bifactor models address this concern by separating out these distinct variance components (Aitken et al., 2020; Constantinou et al., 2019), though assumptions tied to these models may be too strict or unrealistic to use with developmental data (e.g., measurement occasions are assumed to experience equivalent change).

Lastly, the types of cross-sectional models used in prior studies can pose other drawbacks for testing mutualistic processes if the data reflects both positive and negative interactions that can cancel out at the latent level. For instance, in the case of the cross-sectional bifactor model, changes in *p* may appear stagnant if some individuals display broader patterns of symptoms that become increasingly specific over time (e.g., higher levels of *p* lead to narrower symptom expressions), while others show a small range of symptoms that gradually expand throughout development (McElroy, Belsky, et al., 2018). Consequently, methods with developmentally appropriate assumptions that can adequately tease apart between- and within-person dynamics are needed to provide a more accurate investigation of mutualism theory and the *p*-factor.

### The present study

The aim of the present study is to provide a rigorous and more theoretically appropriate test of dynamic mutualism theory by investigating its ability to explain the development of the *p*-factor in a racially diverse, population-based sample of girls who reported annually on various internalizing and externalizing pathologies from ages 14–21.^2^ Our decision to investigate mutualism theory during this developmental window is twofold: first, evidence strongly suggests that escalations in psychopathology tend to occur around early to mid-adolescence (Dalsgaard et al., 2020; Kessler et al., 2005), making this timeframe a reasonable starting point for examining mutualistic processes. This is especially true considering the evidence that suggests females have a higher propensity to develop psychopathology during adolescence compared to their male counterparts (Hayward, 2003; M. Martel, 2013; Ullsperger & Nikolas, 2017). Second, prior research has indicated that the transition from adolescence to young adulthood is one of the most sensitive periods of development due to the significant diversity in life paths, and widespread biological, psychological, and social role changes (e.g., Cicchetti & Rogosch, 2002; Schulenberg et al., 2004; Schulenberg & Zarrett, 2006; Zarrett & Eccles, 2006). The *p*-factor literature, however, has primarily focused on the development of psychopathology during childhood and/or early adolescence (Carragher et al., 2016; Deutz et al., 2020; Lahey et al., 2015; Olino et al., 2018; Patalay et al., 2015; Pettersson et al., 2018; Sallis et al., 2019; Snyder et al., 2017), neglecting important transitions that occur later in development.

In the present study, we tested predictions of dynamic mutualism theory by translating its core assumptions into three statistical models, including longitudinal bifactor models, random-intercept cross-lagged panel models (RI-CLPMs), and network models. While these statistical approaches are not intended to be directly compared, when taken together, results across models offer a detailed description of whether mutualistic processes are supported in the data. Evidence favoring dynamic mutualism theory was based on a series of statistical tests, in which we hypothesized that (1) the bifactor model would reveal a robust general *p*-factor that increased in strength and variance accounted for with age, (2) *p* and/or the internalizing-externalizing factors would have significant, mostly positive bidirectional effects at the between- and within-person levels,^3^ (3) including these bidirectional effects between *p* and the internalizing and externalizing factors in the bifactor models, or between internalizing and externalizing in the RI-CLPMs, would significantly improve model data fit, and (4) associations between internalizing and externalizing indices would strengthen with age, as evidenced by increasing estimates of centrality, edge weights, and small-worldness in the network models.

## Method

### Sample and procedure

Data were obtained from the Pittsburgh Girls Study (*N* = 2450), a prospective longitudinal study conducted in an urban setting in the greater Pittsburgh area (Keenan et al., 2010). Following an enumeration of the city of Pittsburgh, low-income neighborhoods were intentionally oversampled between 1999–2000 to increase the prevalence of girls’ externalizing behavior. Out of the 2875 eligible families, 85% agreed to study participation, resulting in a sample of 2450 girls divided between four age-specific cohorts (ages 5–8). Slightly over half of girls were African American/Black (52.92%), 41.17% were White, and 5.92% identified as a different race or multiracial (for more details on sampling procedures, see Hipwell et al., 2002).

The present analyses utilized eight consecutive annual waves of data (Waves 7–14) that spanned across ages 14–21. Data with sufficient cases were included in the analyses and resulted in a total sample of 2339 girls. At age 14, Wave 7 served as the initial data collection period for cohort 8 and Wave 10 served as the initial data collection period for cohort 5. The average sample retention rate across Waves 7–14 was high (87.26%), with the retention rate for each wave ranging from 86.25%–91.3%.

Attrition analyses, based on logistic regressions, suggested that families who did not receive public assistance or girls who identified as White at Wave 1 were significantly more likely to have incomplete data at age 21. Likewise, missingness at age 21 was significantly related to single-parent status, such that girls who were from a single-parent household at Wave 1 were more likely to remain in the study compared to girls who lived in a two-parent household. Other demographic variables at Wave 1, such as parental education and ethnicity, were not statistically different between those with and without missing data at age 21.

#### Procedure

Study procedures were approved by the University of Pittsburgh Human Research Protection Office. Caregivers provided written informed consent for study participation and girls provided assent until age 18, at which time girls then provided their own informed consent. Computerized assessments were completed separately by girls and their caregivers and families received monetary compensation for their participation (Hipwell et al., 2002).

### Measures

Self-report measures capturing common internalizing and externalizing disorders and related constructs were used to assess for symptoms of attention-deficit hyperactivity disorder (ADHD), conduct disorder and antisocial personality disorder traits (CD/ASPD), generalized anxiety disorder (GAD), major depressive disorder (MDD), oppositional defiant disorder (ODD), and past year frequency of alcohol, marijuana, and tobacco use. Internal consistency was evaluated based on McDonald’s (1999) coefficient Omega (ω), which serves as a more practical index of scale reliability compared to Cronbach’s alpha (Dunn et al., 2014). Reliability across measures generally fell within an acceptable range for research purposes (ω > .70; Hayes & Coutts, 2020) and can be found in Table 1.

The Adolescent Symptom Inventory-4 (ASI-4; Gadow & Sprafkin, 1999) and Adult Self-Report Inventory-4 (ASRI-4; Gadow et al., 2004) measured symptoms of ADHD, CD/ASPD traits, MDD, and ODD. CD traits were measured from ages 14–17 and were substituted with measures of ASPD traits from ages 18–21. The ASI-4 and ASRI-4 are rated based on past year symptoms using a 0–3 Likert scale with response choices: *never*, *sometimes*, *a lot*, and *all the time*.

The Nicotine, Alcohol and Drug Use scale (NADU; adapted from Pandina et al., 1984) was used to assess the frequency of past year alcohol, marijuana, and tobacco use (separately for each substance). The NADU is rated on an 8-point scale, where a “0” denotes *no past year use*, and a “7” signifies use of the substance *every day or more than once a day*. Alcohol use was defined as any consumption of beer, wine, or liquor. As a general proxy for past year substance use, a composite Substance Use score was computed at each age to represent the average frequency of alcohol, marijuana, and tobacco use. Reliability for this scale was generally lower compared to other scales, which may in part reflect the smaller number of scale items used to calculate reliability compared to other measures.

GAD was assessed using the Screener for Child Anxiety Related Emotional Disorders (SCARED; Birmaher et al., 1997) from ages 14–17 and was replaced by the ASRI-4 (Gadow et al., 2004) and one item from the UCLA Loneliness Scale (ULS-20; D. Russell et al., 1980; D. W. Russell, 1996) from ages 18–21. The SCARED is a self-report measure designed to screen for childhood anxiety disorders, such as GAD, separation anxiety disorder, panic disorder, and school phobia. Items are rated on a 3-point Likert scale consisting of the choices: *not true or hardly ever true*, *sometimes true*, and *very true*, respectively. Starting at age 18, girls were administered the anxiety module of the ASRI-4 instead of the SCARED screener. The ASRI-4 and the SCARED have considerable overlap, though the ASRI-4 focuses on generalized anxiety symptoms and consists of 15 questions that are rated on a 0–3 scale.

To ensure a fair comparison of anxiety symptoms across age, a subset of items that best represented GAD was selected from the SCARED and ASRI-4 measures, respectively. Items were matched based on wording and content, yielding a total of eight items from the SCARED, seven items from the ASRI-4 anxiety subscale, and one item from the ULS-20. The ULS-20 item, “is shy,” was specifically added as a parallel to the SCARED item, “I’m shy with people I don’t know well,” and was included with the other ASRI-4 items. Due to differences in Likert scaling between SCARED and ASRI-4 measures, retained anxiety items were re-scaled to be on the same metric using the proportion of maximum scaling method (Little, 2013). Reliability for the created GAD measures were within an acceptable range.

### Data analytic plan

Dynamic mutualism theory was examined by translating its fundamental assumptions into three statistical models. In a recent update of the mutualism model, van der Maas et al. (2017) outlined a comprehensive network model of intelligence that incorporated four primary mechanisms to explain the development of cognitive ability: mutualistic coupling between lower-level cognitive processes, differences in centrality across cognitive processes (e.g., some processes may be more central than others, thereby influencing growth or development at a higher rate), sampling in cognitive test scores, and multiplier effects that are routed through the environment. The authors discuss the utility of network analysis in testing dynamic mutualism, though do not explicitly state that this analytical technique should be the only approach used to examine mutualism theory (van der Maas et al., 2017). As such, the estimated statistical models in the present study were selected based on prior developmental psychopathology and/or intelligence research that has demonstrated the utility of bifactor models, RI-CLPMs, and network models for surveying mutualistic processes (Hofman et al., 2018; Kan et al., 2019; Kievit et al., 2019; McElroy, Belsky, et al. 2018; Murray et al., 2016).

#### Measurement invariance

Longitudinal measurement invariance was assessed in the best fitting bifactor model and RI-CLPM to gauge whether the underlying meaning of the different internalizing and externalizing constructs was interpreted consistently across development (Widaman et al., 2010). More information about these procedures and results are presented in the supplemental materials (see Table S3).

#### Longitudinal bifactor models

Confirmatory bifactor models were estimated using the *lavaan* package in R-Studio (Rosseel, 2012) and included a general *p*-factor and two specific factors reflecting the internalizing and externalizing domains. Internalizing and externalizing composite scores (i.e., sum scores) for each measure served as a proxy for construct/symptom severity and were fixed to load on either the internalizing or externalizing factor in addition to *p*. GAD and MDD were fixed to load on internalizing, while ADHD, CD/ASPD traits, frequency of substance use, and ODD were fixed to the externalizing factor. An average severity score was computed for CD/ASPD traits rather than a sum score to account for differences in the number of items used to measure CD and ASPD symptoms.

Bifactor models were estimated using robust maximum likelihood (MLR) to account for any data non-normality (Satorra & Bentler, 1994) and were identified by fixing factor variances to 1 with a mean of 0. Missing data due to sample attrition were handled with Full-Information Maximum Likelihood (FIML), which performs equally well, if not better, than other missing data techniques (e.g., multiple imputation; Larsen, 2011). Model fit was judged based on the AIC and robust variants of the CFI, TLI, and RMSEA that are corrected for non-normality (Brosseau-Liard & Savalei, 2014; Savalei, 2018). CFI and TLI ≥ .90 and RMSEA ≤ .06 were indicative of adequate model fit (Hu & Bentler, 1999; Little, 2013; Schermelleh-Engel et al., 2003). The chi-square goodness of fit statistic was reported, though was not of primary interest due its oversensitivity in larger samples (Floyd & Widaman, 1995). As a final check, we also estimated exploratory bifactor models to examine possible sources of misfit and cross-loadings (Marsh et al., 2014; Morin et al., 2020). Estimation procedures and model results are reported in the supplemental materials (see Tables S1 and S2).

Parallel to other research (e.g., Greene & Eaton, 2017; Olino et al., 2018; Snyder et al., 2017), we estimated two bifactor variants that provided information on between- and within-factor stability. Whereas the first model included only autoregressive paths for each factor (e.g., *p* at age 14 predicted itself at age 15), the second model introduced cross-lagged paths between *p* and the specific factors over time. In line with mutualism theory, we expected the bifactor with cross-lagged paths to demonstrate positive associations between the specific factors predicting *p*, but not vice versa.^4^ This model was in turn predicted to statistically outperform the bifactor with only autoregressive paths.

#### Factor strength, reliability, and replicability

Factor strength, model-based reliability, and construct replicability for *p* and the specific factors was quantified by calculating the explained common variance (ECV), Omega total (ω), Omega Subscale (ω_S_), Omega Hierarchical/Hierarchical Subscale (ω_*HS*_/ω_*HS*_), Relative ω, and Hancock and Mueller’s (2001) *H* construct replicability index (Reise, 2012; Rodriguez et al., 2016a, 2016b). All indices were calculated using the Microsoft Excel Bifactor Indices Calculator (Dueber, 2017) and were derived from the confirmatory and exploratory bifactor models at each age.

The ECV provides an index of factor strength and is the proportion of common variance explained by a given factor (Reise et al., 2013, Rodriguez et al., 2016a, 2016b; Sijtsma, 2009; Stucky & Edelen, 2014). ω is an index of model-implied reliability that returns the proportion of common variance across all factors relative to the total variance. ω_*S*_ is related to ω, though returns the proportion of variance in observed subscale scores that is explained by the general factor and a given specific factor. Of particular interest, ω_*H*_ is the percentage of systematic variance in raw total scores that is attributable to the general factor after controlling for the influence of the specific factors. ω_*HS*_ is the specific factor version of ω_*H*_ and reflects the percentage of variance attributable to a specific factor after accounting for the variance explained by the general factor (McDonald, 1999; Reise et al., 2013; Rodriguez et al., 2016b; Zinbarg et al., 2005). Relative ω reflects the proportion of reliable variance in total (or subscale) scores that are attributable to the general factor or a specific factor, respectively (Rodriguez et al., 2016a). Lastly, the construct replicability index (*H*) assesses the replicability of the modeled factors by evaluating how well a latent variable is defined by its indicators. Values of *H* ≥ .70 indicate that the factor is well-represented by its respective items and is likely to be replicable by other studies (Hancock & Mueller, 2001, Rodriguez et al., 2016a, 2016b).

To be consistent with mutualism theory, estimates of ω_*H*_ and ECV for the *p*-factor are predicted to progressively increase over time, with *p* capturing substantially more variance compared to the specific factors. Support against mutualism, in contrast, is evidenced by stagnant estimates of strength and/or variance accounted for by the *p*-factor over time. In determining whether the strength and/or variance explained by *p* meaningfully increased, Wald tests were used to statistically evaluate if changes in ω_*H*_ or ECV were significant. In other words, because both ω_*H*_ and ECV can be calculated from the estimated factor loadings, we constrained loadings on *p* to be equal at the first and last time point (i.e., ages 14 and 21) and used Wald tests to assess for any significant differences. If Wald tests were significant, factor loadings at age 14 and 21 were inspected to determine the direction of this difference.

#### Random-intercept cross-lagged panel models (RI-CLPMs)

The RI-CLPM allowed the relationships between internalizing and externalizing to be examined at the within-person level, independent of the *p*-factor. The RI-CLPM can be thought of as an extension of the traditional cross-lagged panel model, except most parameters are interpreted at the within-person level by including a random-intercept factor (Berry & Willoughby, 2017; Hygen et al., 2020). In other words, after accounting for the more stable between-person differences captured in the random-intercepts, autoregressive paths reflect the extent that deviations above or below one’s personal average (i.e., expected score) carry-over into the next measurement occasion. Similarly, cross-lagged paths quantify whether person-specific deviations in one domain predict comparable deviations in a separate domain, while within-time factor correlations capture the degree that person-specific deviations at the same measurement period are related between domains (Berry & Willoughby, 2017; Hamaker et al., 2015).

RI-CLPMs were constructed in the *lavaan* package (Rosseel, 2012) with MLR and FIML estimation and were evaluated using the same goodness of fit criteria as the bifactor models. However, the RI-CLPMs did not include a general *p*-factor, as the purpose of this model was to determine if interactive effects were present between the internalizing and externalizing factors that would otherwise be subsumed by *p*. The first model served as a baseline model where autoregressive paths for the internalizing and externalizing factors were freely estimated but cross-lagged paths were constrained to zero. This model was expected to provide the poorest fit to the data if predictions of mutualism are supported. Next, we estimated two unidirectional models (i.e., internalizing predicting change in externalizing or vice versa), which was followed by the bidirectional model that served as a proxy for mutualism. Chi-square difference tests and the AIC were used to compare the nested RI-CLPMs, and parameters from the superior fitting model were further inspected. Support for dynamic mutualism theory was based on whether the bidirectional RI-CLPM outperformed all other models, with the internalizing and externalizing factors expected to have mostly positive, significant cross-lagged associations over time. Insignificant and/or pre-dominantly negative associations between domains were interpreted as evidence against mutualism theory.

#### Network models

As a final probe of dynamic mutualism, we used network analysis to examine reciprocal associations between internalizing and externalizing constructs independent from their latent domains. In doing so, we estimated weighted, unregularized networks as Gaussian Graphical Models (GGM; Lauritzen, 1996) using the *bootnet* package (i.e., “ggmModSelect”) in R-studio (Epskamp, 2015). GGMs were purposely not regularized, as unregularized estimation procedures are shown to outperform regularized networks when sample sizes are large and a small set of nodes are estimated (Foygel & Drton, 2010; Friedman et al., 2008; Williams & Rast, 2020; Williams et al., 2019). Under GGM estimation, a graphical LASSO algorithm is implemented that iteratively re-estimates the network without regularization using maximum likelihood estimation. This algorithm yields the most parsimonious model by adding or removing edges until the extended Bayesian Information Criteria no longer improves (Isvoranu & Epskamp, 2021).

To mirror the separation of processes in the RI-CLPMs, networks were estimated at the between- and within-person levels and missing data was handled with FIML. Whereas between-person networks explain the covariance patterns of stationary means *across* individuals, within-person networks detail the covariances of stationary means *within* individuals (Epskamp, Waldorp, et al., 2018). Within-subject networks were constructed using an approach outlined by Costantini et al. (2019), where each subject’s grand mean is computed per node and subtracted from the subject’s observed score at a given age.

Akin to other developmental work (e.g., McElroy, Shevlin, et al., 2018), we estimated three cross-sectional networks that were equidistant in time. This resulted in two networks (i.e., one between and one within-person) estimated at ages 14, 17, and 20. Age 20 was selected as the final estimated age rather than age 21 to ensure network comparisons were equally spaced. Due to the computational complexity required to estimate a symptom-level network, nodes were based on the same composite scores used in the bifactor models and RI-CLPMs. Average substance use frequency was the exception, and each substance was modeled as its own node.^5^

The *bootnet* package was used to assess the accuracy of the edge weights by calculating 95% confidence intervals (CIs) around the edges with non-parametric bootstrapping (Epskamp, 2015). Centrality stability was subsequently inspected via a case-drop bootstrapping approach, which allowed the correlation stability coefficient (CS-coefficient) to be subsequently estimated. The CS-coefficient reflects the proportion of cases that can be dropped to maintain a correlation of at least .70 between the original and bootstrapped network. CS-coefficients above .70, .50, and .25 indicate excellent, good, and fair stability, respectively (Epskamp, Borsboom, et al., 2018). In addition, bootstrapped difference tests were conducted for centrality metrics and edges to gauge whether these indices significantly differed from one another. The difference test results can be found in the supplemental materials (Figures S3–S6).

Node importance was judged by estimating centrality measures of closeness, betweenness, and expected influence (EI). Closeness refers to the average shortest path length between different pairs of nodes and quantifies the *indirect* influence of a given node. Betweenness is the number of times a node falls on the shortest path between two other nodes. Thus, nodes with high betweenness are often interpreted as bridges that foster connections between other nodes (Costantini et al., 2015, 2019; Newman, 2010; Opsahl et al., 2010). EI is similar to the strength centrality metric and supplies an index of how influential a node is in the entire network structure. Unlike strength, however, EI takes into account both positive and negative edge weights in its calculation (McNally, 2016; Robinaugh et al., 2016).

The small-worldness index (SWI) was obtained using the *qgraph* package (Epskamp et al., 2012) and is computed based on the average shortest path length and overall transitivity of the network (Newman, 2010). Networks characterized by high degrees of small-world properties have SWI values greater than 1 (with stricter cutoffs of 3 or more; Humphries & Gurney, 2008) and are sensitive to fluctuations in the network, such that changes in a single node are more likely to influence other nodes in the network (Borsboom et al., 2011). If predictions of mutualism are supported, the SWI is expected to exceed 1 and gradually increase throughout development. If the SWI is instead found to be small or weaken over time, this suggests that the network structure is becoming sparser with age and is inconsistent with mutualism theory.

#### Network comparisons

The *NetworkComparisonTest* package in R-studio was used to test whether networks differed in structure or global connectivity (van Borkulo et al., 2016). Structural invariance is reflected in the *M* test statistic and is the maximum difference across edges between two networks. Global invariance compares differences in the overall strength (i.e., the absolute value of the sum of edges) of two networks and is reflected in the *S* test statistic (Opsahl et al., 2010; van Borkulo et al., 2017). If significant differences at the structural or global level were observed, edge weight difference tests with a holm *p*-value adjustment were used to determine which edges statistically differed between networks (van Borkulo et al., 2017). Although NCTs can be used with dependent samples, the algorithm for dependent samples is still undergoing validation (van Borkulo et al., 2016). Therefore, NCTs were supplemented by correlating edges and centrality metrics across networks to further gauge differences in the network structures.

## Results

Means, standard deviations (SDs), and reliability for each of the internalizing and externalizing measures are presented in Table 1. In brief, the best fitting bifactor and RI-CLPM were found to be partially invariant at the scalar level, with the bifactor demonstrating greater variability in factor loadings compared to the RI-CLPM. Globally, violations of measurement invariance appeared to be small and were mostly attributable to fluctuations in CD/ASPD traits and mean-level changes in the average frequency of substance use over time. ODD similarly exhibited some degree of metric non-invariance in the bifactor model, with its loadings on *p* decreasing over time. Additional information on measurement invariance procedures and results can be found in the supplemental materials (Table S3).

### Longitudinal bifactor models

#### Exploratory models

Standardized factor loadings from the exploratory models are reported in the supplemental materials (Table S1). Cross-loadings for internalizing and externalizing were generally small and fell below .20 across age. Estimates of factor strength, reliability, and replicability are similarly presented in the supplementals (Table S2). Estimates of factor strength and reliability for the *p*-factor were similar between confirmatory and exploratory models; however, exploratory models suggested that the variance accounted for by the specific factors was substantially weaker relative to the confirmatory models (described below).

#### Strength, reliability, and construct replicability of *p* and the specific factors

Factor strength, reliability, and construct replicability based on confirmatory bifactor models at each age are reported in Table 2. ECV and ω_*H*_ suggested that the strength and proportion of variance explained by *p* steadily increased throughout development, reaching its peak value at age 21 (ECV = .74; ω_*H*_ = .76). Relative ω for *p* similarly reached its peak value at age 21 (relative ω = .86), suggesting that 86% of the reliable variance in total scores can be attributed to the *p*-factor (Rodriguez et al., 2016b). Construct replicability for the *p*-factor was also high, with *H* above recommends cutoffs of ≥.70 at all ages. After controlling for the influence of the *p*-factor, ω_*HS*_ indicated that the variance attributable to the specific factors was substantially weaker. Whereas internalizing accounted for a greater proportion of variance at each age relative to externalizing (ω_*HS*_ = .21–.35), internalizing steadily decreased in the amount of variance it explained over time while externalizing remained stable (ω_*HS*_ = .18–.22).

In determining whether these increases in strength and/or reliable variance for the *p*-factor were statistically meaningful, equality constraints were imposed on each respective factor loading on *p* at the first and last time point (i.e., age 14 and age 21). Significant Wald tests were documented for ADHD (Wald test Σ^2^ (1) = 49.78, *p* < .001), CD/ASPD traits (Wald test Σ^2^ (1) = 35.65, *p* < .001), MDD (Wald test Σ^2^ (1) = 37.40, *p* < .001), and ODD (Wald test Σ^2^ (1) = 149.67, *p* < .001). In contrast, GAD (Wald test Σ^2^ (1) = 0.91, *p* = .34) and substance use frequency (Wald test Σ^2^ (1) = 1.20, *p* = .27) resulted in non-significant differences between ages. Inspection of factor loadings suggested that ADHD, CD/ASPD, and MDD displayed stronger loadings on *p* over time, while loadings for ODD marginally decreased by age 21.

#### Model fit and factor stability

Factor loadings and standard errors for the longitudinal bifactor models are presented in the supplemental materials (Table S4). The bifactor model with only autoregressive paths provided excellent fit to the data (SB-χ^2^ (*df*) = 1939.79 (843), *p* < .001; R-CFI = .98; R-TLI = .97; R-RMSEA [90% CI] = .026 [.025–.028]; AIC = 322,793), with most loadings on the *p*-factor significant over time (*p* < .001). We next compared this model to a similar bifactor structure that included cross-lagged paths between *p* and the specific factors. This bifactor variation with cross-lagged paths had excellent fit to the data (SB-χ^2^ (*df*) = 1633.08 (801), *p* < .001; R-CFI = .98; R-TLI = .98; R-RMSEA [90% CI] = .023 [.022–.025]; AIC = 322,444), with the inclusion of these paths significantly improving model fit (Δχ^2^ (Δ*df*) = 241.21 (42), *p* < .001).

Estimates of within- and between-factor stability (i.e., autoregressions and cross-lags, respectively) can be found in Table 3. When cross-lagged effects were included, the *p*-factor was determined to have weaker temporal stability at later ages, with strong stability throughout adolescence (β = .29–.72, *p* < .01). Autoregressive paths for externalizing were larger, on average, compared to both *p* and internalizing, with these effects generally increasing with age (β = .48–.80, *p* < .001). Conversely, temporal stability for internalizing was more variable and tended to decline with age (β = .51–.86, *p* < .05). Autoregressive paths were mostly significant, apart from internalizing at age 17 predicting internalizing at age 18 (β = .88, *p* = .11).

In line with dynamic mutualism theory, the specific factors were found to significantly predict *p* at several ages, with within-factor stability (i.e., autoregressions) for *p* declining once cross-lagged paths were specified. Cross-lags were especially pronounced for internalizing predicting *p*, such that internalizing significantly predicted *p* at each age with these effects increasing over time (β = .10–.46, *p* < .05). In contrast, externalizing significantly predicted *p* at age 18 (β = .26, *p* < .01) and age 19 (β = .42, *p* < .01), and was itself predicted by *p* at ages 17–19 (β = .21–.42, *p* < .05) and age 21 (β = .20, *p* < .05). Significant cross-lags were further documented for internalizing negatively predicting future levels of externalizing between ages 15–17 (β = −.27 to −.14, *p* < .05) and again at age 20 (β = −.24, *p* < .05), though externalizing in turn only predicted internalizing at age 15 (β = −.23, *p* < .001).

### Random-intercept cross-lagged panel models

#### Model fit and comparisons

Fit statistics for the RI-CLPMs are presented in Table 4 and suggested that all model variations demonstrated excellent fit to the data (CFI and TLI > .95, RMSEA < .08). Although R-CFI, R-TLI, and R-RMSEA produced equivalent estimates across models, chi-square difference tests and the AIC suggested that the mutualism model with bidirectional effects provided the best fit to the data. Parameter estimates for this model are reported in Table 5.

#### Interpretation of parameter estimates from the mutualism model

Parameters from the mutualism RI-CLPM indicated significant between-person variability as evidenced by the random-intercept factor variances. Stated differently, variances of the random-intercepts were found to statistically differ from 0, indicating significant between-person variability for both internalizing (σ^2^ = 5.23, *p* < .001) and externalizing (σ^2^ = 10.07, *p* < .001) domains. These between-person components were also significantly correlated (σ^2^_B_: *r* = .78, *p* < .001), implying that girls who scored above average on internalizing were more likely to score above average on externalizing throughout development.

At the within-person level, significant autoregressive paths were documented for internalizing across age (β = .29–.58, *p* < .05), providing some evidence for within-person carry-over effects. In other words, girls who deviated from their expected score in internalizing at a given age (i.e., girls who scored either above or below their personal average) were more likely to show similar deviations in internalizing at subsequent ages. Significant autoregressive effects also emerged for externalizing but were less stable compared to internalizing and were only significant from ages 15–19 (β = .20–.60, *p* < .05). Thus, compared to the autoregressions in the bifactor model that indicated externalizing to be the most stable factor over time, within-person estimates found the opposite pattern of results.

In contrast, cross-lagged effects were mostly non-significant for internalizing predicting change in externalizing throughout adolescence, implying that person-specific deviations in externalizing symptoms were not dependent upon prior deviations in internalizing. This pattern, however, did shift by adulthood, such that internalizing significantly predicted within-person change in externalizing at ages 20 (β = .27, *p* < .05) and 21 (β = .73, *p* < .01). Relatedly, cross-lagged paths from externalizing predicting internalizing were negative during adolescence but became positive, albeit non-significant, by early adulthood. Specifically, externalizing significantly predicted within-person change in internalizing at age 15 (β = −.23, *p* < .01), age 16 (β = −.16, *p* < .05), and age 18 (β = −.28, *p* < .05). This indicated that within-person deviations in internalizing, at least during these ages, can be predicted by an individual’s prior deviation from their expected score in externalizing (e.g., girls who reported above average symptoms of externalizing, relative to their personal average, were more likely to report fewer internalizing symptoms a year later). Within-time factor correlations were also positive and linearly increased with age, implying that girls who scored above or below their personal average on internalizing showed comparable deviations in externalizing at the same measurement occasion.

As an added check, we also examined the extent that effects in the mutualism RI-CLPM changed when between-person variability was not controlled for by constraining the variances and covariances of the random-intercept factors to 0. This resulted in a nested model under the RI-CLPM that is equivalent to the cross-lagged panel model (CLPM; Hamaker et al., 2015). Constraining the random-intercept variances and covariances resulted in significantly poorer fit to the data based on the chibar-square test^6^ *P*(*x*^2^ = 6.46, *p* < .001), which further supported that these domains were characterized by meaningful differences across individuals (Stoel et al., 2006). Intriguingly, when these between-person effects were not directly accounted for, autoregressive paths became more pronounced for internalizing (β = .77–.89, *p* < .001) and externalizing (β = .63–.78, *p* < .001). Cross-lagged effects from externalizing to internalizing also decreased in frequency, such that externalizing only predicted internalizing at age 15 (β = −.16, *p* < .01) but was positively predicted by internalizing at age 15 (β = .09, *p* < .05), age 17 (β = .11, *p* < .01), and age 18 (β = .12, *p* < .05).

### Network models

**Figure 1.**
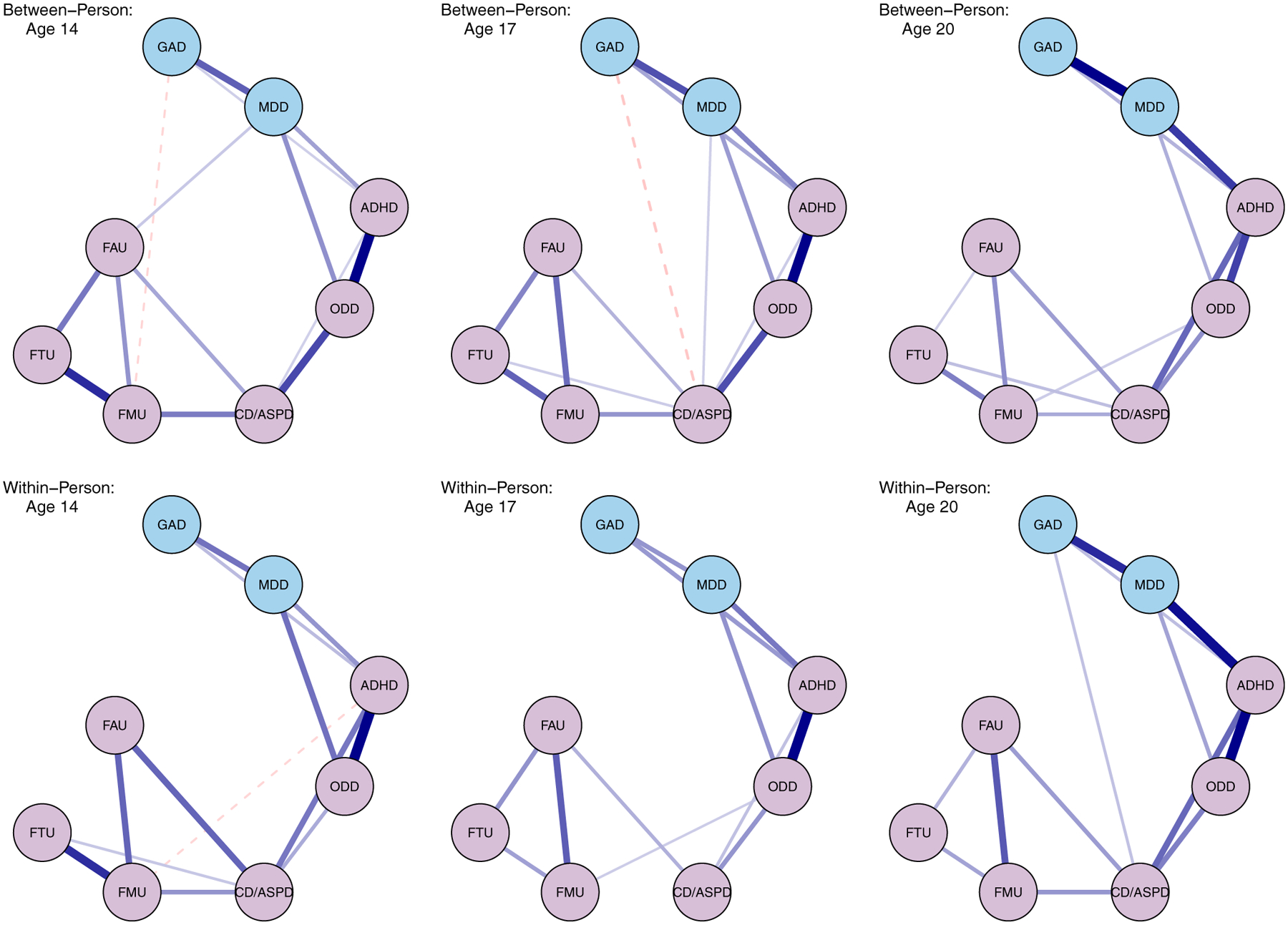
Between- and within-person network graphs by age. Nodes and edges are represented by circles and lines, respectively. Thicker lines indicate stronger associations between two nodes after controlling for all other associations in the network. ADHD = attention-deficit hyperactivity disorder; CD/ASPD = conduct disorder/antisocial personality disorder traits; FAU = frequency of alcohol use; FMU = frequency of marijuana use; FTU = frequency of tobacco use; GAD = generalized anxiety disorder; MDD = major depressive disorder; ODD = oppositional defiant disorder.

Graphs of the between- and within-person networks for ages 14, 17, and 20 can be found in Figure 1. For ease of visual comparison, networks are presented using the Fruchterman and Reingold (1991) graphing algorithm (i.e., the “spring” layout in the *qgraph* package) using the same average layout (Epskamp et al., 2012). More densely connected nodes, which are represented as circles, are concentrated together towards the middle of the graph.

#### Accuracy and stability of networks

Nodes with stronger edges were found to have smaller CIs that did not overlap with zero, implying that these edges were more accurate compared to weaker edges that generally had larger CIs (Epskamp, Borsboom, et al., 2018). For example, node pairs such as ADHD-ODD, GAD-MDD, MDD-ADHD, and frequency of alcohol use with other substance use nodes generally had smaller CIs that did not overlap with zero. In contrast, node pairs such as MDD-CD/ASPD tended to have weaker edges with wider CIs (see Figure S1).

Centrality stability for the between-person networks was fair for betweenness (CS-coefficients > 0.50), with good metric stability found for closeness and EI (CS-coefficients > 0.70). Within-person networks suggested that stability was poor for betweenness (CS-coefficients < 0.25), though ranged from adequate to good for closeness (CS-coefficients > 0.50–0.70) and EI (CS-coefficients > 0.70). Estimates of betweenness were thus not interpreted for within-person networks, and node centrality was evaluated based on closeness and EI. More information on the accuracy and stability of the networks are reported in the supplemental materials (Figures S1–S6).

#### Between-person networks

Nodes associated with the internalizing or externalizing domains generally clustered together, with substance use nodes forming their own cluster separate from the externalizing nodes. Several positive edges were documented between the various internalizing and externalizing nodes, which was consistent with small-worldness estimates above 1 – but not above stricter cutoffs of 3 (SWI_14_ = 1.07; SWI_17_ = 1.27; SWI_20_ = 1.47).

**Figure 2.**
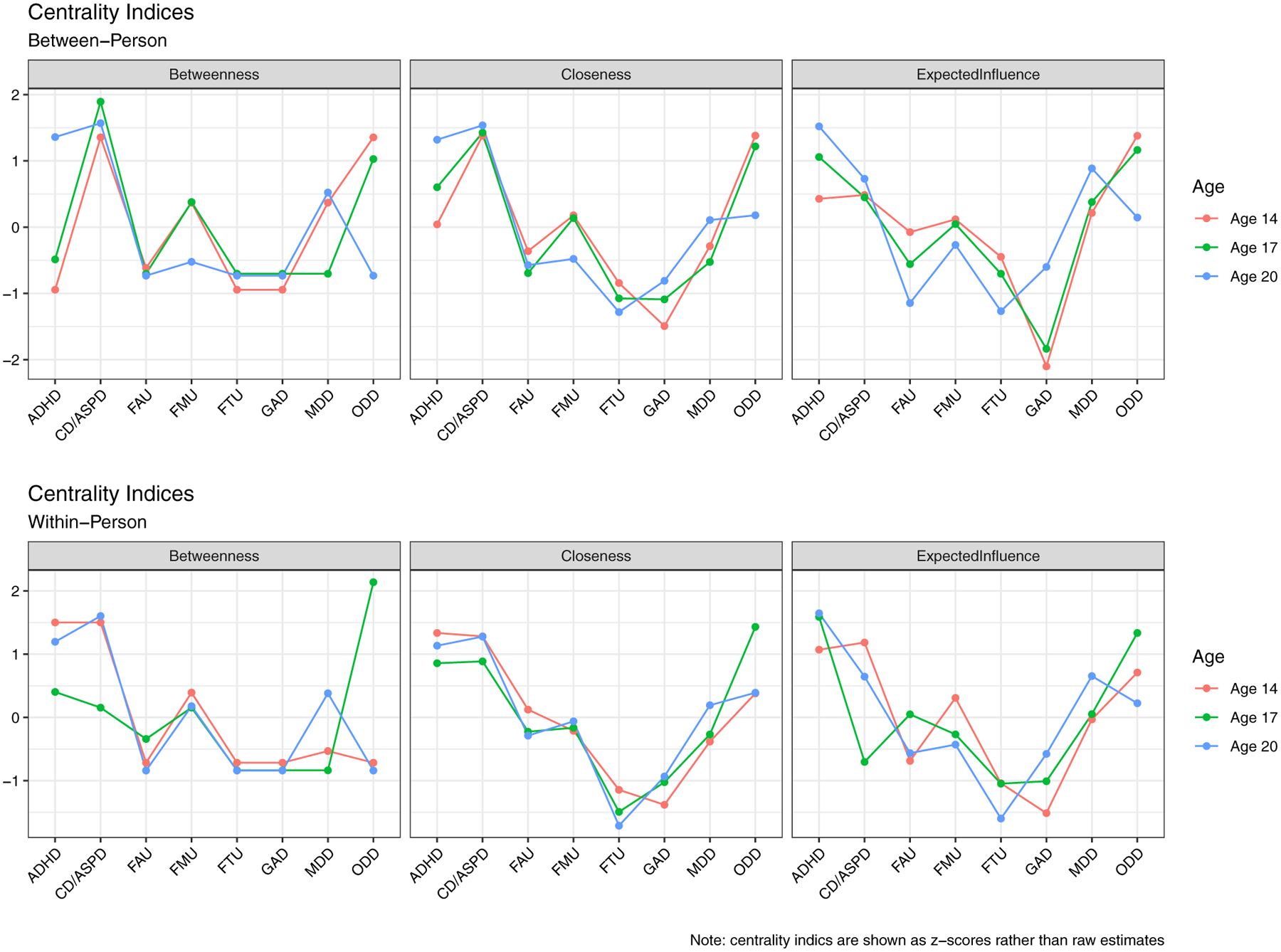
Centrality for between- and within-person networks by age. ADHD = attention-deficit hyperactivity disorder; CD/ASPD = conduct disorder/antisocial personality disorder traits; FAU = frequency of alcohol use; FMU = frequency of marijuana use; FTU = frequency of tobacco use; GAD = generalized anxiety disorder; MDD = major depressive disorder; ODD = oppositional defiant disorder.

Standardized centrality estimates by age are displayed in Figure 2. Correlations between centrality indices ranged from moderate to large and can be found in the supplemental materials (Table S5). ADHD, CD/ASPD, and ODD tended to be more central in the network, while GAD and substance use related nodes were least central overall (Figure 2). ADHD was determined to be the most central node overall due to its positive associations with other internalizing and externalizing nodes, high estimates across the different centrality measures, and its centralized position in the network structure (Figure 1).

Edge weights were found to generally strengthen over time apart from ODD and substance use nodes. ADHD had the largest number of positive edges and was significantly related to CD/ASPD, ODD, GAD, and MDD across age. Pearson correlations for the estimated edges were largest between ages 14 and 17 (*r* = .94), followed by ages 17 and 20 (*r* = .85), and ages 14 and 20 (*r* = .79). Structural differences and edge weight differences were non-significant between ages 14 and 17 (*M* = 0.12, *p* = .30), though were significant between ages 14 and 20 (*M* = 0.19, *p* < .001), and ages 17 and 20 (*M* = 0.19, *p* < .01). Results of the edge weight difference tests indicated that MDD-GAD, MDD-ADHD, and ADHD-CD/ASPD edges significantly increased with age, while ODD-ADHD, ODD-CD/ASPD traits, marijuana and tobacco use, and alcohol and tobacco use edges decreased over time (see Table S7 in the supplemental materials). Notwithstanding the reported structural non-invariance and increases in small-worldness, the global connectivity of the network (i.e., sum of all edges) remained stable over time. That is to say that while the broader levels of psychopathology were consistent, the structural patterns and manifestations of these pathologies appeared to fluctuate to some degree.

#### Within-person networks

Networks at the within-person level had comparable clustering patterns to the between-person structures (see Figure 1). Further, although the SWI of the within-person networks was characterized by sharper increases, both between- and within-person structures reached similar levels of small-worldness by age 20 (SWI_14_ = 1.16; SWI_17_ = 1.47; SWI_20_ = 1.44). This suggested that the density of the network increased throughout development, such that the activation of one node was more likely to have downstream effects on other nodes in the network (Borsboom et al., 2011).

Centrality estimates also mirrored the between-person networks, such that ADHD, ODD, and CD/ASPD tended to be the most influential in the network, followed by MDD at later ages (Figure 2). Correlations amongst centrality measures, on average, were greatest for ages 14 and 20, with weaker correlations between ages 14 and 17 (see supplemental Table S6). Analogous to the between-person networks, positive edges were found between GAD-ADHD, MDD-ADHD, and MDD-ODD at all ages, implying that ADHD may serve as a bridge node between other internalizing and externalizing nodes.

Edges were also highly correlated across age (Ages 14 and 17: *r* = .82; Ages 17 and 20: *r* = .87; Ages 14 and 20: *r* = .84), though exhibited greater oscillations relative to the between-person networks. For instance, despite most edges in the between-person networks progressively increasing with age, several edges in the within-person structures decreased in strength from age 14 to age 17, though increased in magnitude between ages 17 and 20. Likewise, some node pairs, such as GAD and ADHD, were found to have stagnant edges between ages 14 and 17 that sharply increased by age 20. These edge fluctuations were in turn verified by the NCTs, which revealed significant differences in both the structure and global connectivity of the networks across time. Structural differences were most pronounced for ages 14 and 20 (*M* = 0.22, *p* < .001), trailed by ages 14 and 17 (*M* = 0.20, *p* = .002), and 17 and 20 (*M* = 0.15, *p* = .04). In contrast, significant differences in global connectivity were largest between ages 14 and 17 (*S* = 0.58, *p* < .001), followed by ages 14 and 20 (*S* = 0.30, *p* < .01), and ages 17 and 20 (*S* = 0.29, *p* < .001). Significant differences in edge weights were most common for ADHD, CD/ASPD, MDD, GAD, and substance use related nodes (see Table S7 in the supplementals). In combination, these results suggested that the overall connectivity of the network was characterized by both decreases and increases, with the structure of the network similarly changing over time.

## Discussion

The present study evaluated the explanatory power of dynamic mutualism theory in accounting for the developmental trajectories of the *p*-factor from ages 14–21. As research has remained limited in documenting the longitudinal trajectories of *p* from adolescence to young adulthood, we extend previous work by exploring the development and stability of *p* and the internalizing-externalizing factors during this important transitional period.

In efforts to provide a more comprehensive and theoretically compatible test of mutualism theory, we constructed three distinct statistical models to evaluate whether mutualistic processes were supported at the between- or within-person levels. Taken together, the results of the present study offer some support for the role of mutualistic processes in the development of *p*; however, our findings are intended to be preliminary in nature, and do not discount the potential for other processes or mechanisms to influence the development of psychopathology.

Regarding mutualistic processes at the between-person level, the bifactor models found support for a robust general *p*-factor that systematically increased in strength and variance explained with age. Wald tests indicated that these increases were unlikely due to chance, and that *p* and the internalizing-externalizing factors may be characterized by more nuanced dynamics from mid-adolescence to adulthood than previously reported (Castellanos-Ryan et al., 2016; Murray et al., 2016; Snyder et al., 2017). Furthermore, results indicated that the specific factors positively predicted *p* at several ages, with the inclusion of these cross-lagged paths significantly improving goodness of fit. Cross-lag effects tended to strengthen with age, particularly for internalizing predicting *p* and less so for externalizing and *p*. Consistent with mutualism theory, this suggested that symptom expression in a specific area of psychopathology (e.g., internalizing: depression) was associated with greater risk for developing broader symptoms from either domain in the future. Relatedly, our results found the between-person components of the internalizing and externalizing factors to be strongly correlated in the RI-CLPMs. In line with the shared-risk hypothesis (Angold et al., 1999), this indicated that the co-development of internalizing and externalizing symptoms and related behaviors were partially attributable to stable, time-invariant risk factors that are shared across domains.

In terms of within-person associations, we found significant, albeit small, cross-lags between the internalizing and externalizing factors in the bidirectional RI-CLPM. That is to say that after accounting for the other between- and within-person effects, significant cross-lags still emerged, with the bidirectional model statistically outperforming other models. Results indicated that internalizing positively predicted externalizing starting at age 18, though these paths were only significant at ages 20 and 21. Clinically, these findings imply that targeting internalizing symptoms in late adolescence may be effective in preventing co-occurring externalizing problems from developing in adulthood.

In comparison, externalizing was a significant, though negative, predictor of internalizing at several ages throughout adolescence. This suggested that relatively higher levels of externalizing were associated with subsequent decreases in internalizing, highlighting a potential protective effect of externalizing in adolescence. Notably, these negative associations are inconsistent with longitudinal evidence that has reported externalizing to positively predict within-person change in internalizing during early childhood (Oh et al., 2020), and at several points from childhood into adolescence (Flouri et al., 2019). Nonetheless, findings in this area have been mixed and other within-person studies have found externalizing to positively predict internalizing throughout childhood but negatively predict internalizing by early adolescence (Murray et al., 2020; Obsuth et al., 2020). Considering we examined these associations beginning at age 14, it is possible that our results reflect different developmental processes between adolescence and preceding stages of development, such that the positive associations predicted for internalizing and externalizing may be more pronounced in childhood rather than adolescence (e.g., Flouri et al., 2019; Murray et al., 2020; Oh et al., 2020).

Notably, when between- and within-person variance components were not directly separated, negative cross-lagged paths from externalizing to internalizing became less frequent, while positive cross-lags from internalizing to externalizing increased. These results highlight the importance of disentangling more stable between-person processes from within-person dynamics (Hamaker et al., 2015), and are congruent with studies that suggest within-person continuities between internalizing and externalizing are weaker once shared, time-invariant factors are controlled for at the between-person level (Wichstrøm et al., 2017).

Despite some negative associations in the RI-CLPMs, between- and within-person networks found several positive associations between internalizing and externalizing nodes over time. SWI estimates also increased with age and exceeded proposed cutoffs (SWI ≥ 1), suggesting that the network structure became more densely connected throughout development. However, parallel to findings of Sterba et al. (2010), the estimated networks did not indicate a clear pattern of increasing or decreasing associations between internalizing and externalizing nodes. For example, while some internalizing and externalizing pairs increased in strength (e.g., ADHD and MDD), other node associations became weaker over time (e.g., ODD and MDD, FAU and MDD). Edges between ADHD and GAD were one of the few exceptions and remained more consistent, which may shed light on the positive cross-lags found for internalizing predicting externalizing in the RI-CLPM. Several studies have reported similar associations between internalizing symptoms and ADHD (Biederman et al., 2008; McElroy, Shevlin, et al., 2018; Murray et al., 2022; Speyer et al., 2021; Wichstrøm et al., 2017), though ADHD is usually suggested to increase the probability of anxiety and depression rather than the reverse. Yet, it is possible that after accounting for any stable commonalities between ADHD and GAD (i.e., deficits in executive functioning; Mogg et al., 2015), and ADHD and MDD (i.e., genetic overlap; Riglin et al., 2020), the effect of internalizing on ADHD symptoms is more identifiable at the within-person level during this developmental period (Murray et al., 2022).

In sum, the results discussed insofar provide some support for mutualism theory; however, it is important to note that some findings were also inconsistent with dynamic mutualism. First, the bifactor model indicated that *p* was a significant predictor of externalizing in late adolescence, and to a lesser degree, internalizing, which was incongruent with our original predictions. Given that *p* was largely defined by high levels of impulsivity/disinhibition (i.e., ADHD and ODD indicators) and the externalizing factor was mostly characterized by substance use and CD/ASPD traits, one interpretation of this finding is that it reflects links between impulsivity and substance use that are commonly found during mid-adolescence (Gullo & Dawe, 2008; Quinn & Harden, 2013), especially for girls (Kong et al., 2013). Conversely, this may suggest that the relationship between *p* and externalizing during mid-adolescence is better characterized by differentiation-related processes rather than mutualistic processes, at least at the between-person level.

In addition to findings from the bifactor models, the bidirectional RI-CLPM suggested that a large proportion of significant cross-lags between internalizing and externalizing were negative. While these negative effects weakened over time, the fact that earlier periods of development were characterized by greater negative associations is largely inconsistent with a core prediction of dynamic mutualism (i.e., mostly positive associations). Considering several of our results were congruent with mutualism theory, it is possible that some of these mixed findings could reflect a misalignment between the measurements in the current study and the period in which these temporal dynamics truly unfold (Aristodemou et al., 2021). For example, causal interactions between internalizing and externalizing symptoms and/or disorders may be inadequately captured by the present study if these dynamics were stronger before age 14 or after age 21. Equally, it is conceivable that our yearly assessments may fail to capture some of the developing associations between internalizing and externalizing if these dynamics are better represented by more frequent measurements (e.g., weekly, monthly, bi-annually).

Alternatively, and akin to prior conclusions (Aristodemou et al., 2021; McElroy, Belsky, et al., 2018; Murray et al., 2020), these conflicting findings could alternatively be interpreted as evidence for multiple developmental processes to influence the overall trajectories of psychopathology. Seeing as the transition from adolescence to young adulthood is characterized by a multitude of biological, environmental, social, and psychological changes (Feldman et al., 1990; Masten, 2006), it seems reasonable that both mutualistic (e.g., narrow symptom presentations lead to broader expressions of psychopathology), and differentiation processes (e.g., broader forms of psychopathology lead to specific symptom manifestations) could describe the progression of psychopathology at different developmental stages. These underlying processes, in turn, may be influenced by other developmentally relevant distal or proximal factors that were unable to be incorporated in the current models (McLaughlin, 2016). For example, use of alcohol or other drugs among close friends is a robust predictor of adolescent substance use (Cousijn et al., 2018; Glaser et al., 2010), and if included in our analyses, may have led to slightly different conclusions.

Indeed, the potential for external factors to influence the measured trajectories of psychopathology was indirectly supported by the bidirectional RI-CLPM, which found large, positive within-time correlations between the internalizing and externalizing factors that increased over time. This suggested that girls who reported relatively higher levels of symptoms in one domain were found to report similar elevations in the other domain (and vice versa). These correlations could be interpreted as synchronous effects, or as evidence for unmeasured, time-*variant* factors that influence within-person change in both domains simultaneously (Willard et al., 2021). In other words, while the random-intercept factors control for the effect of stable, time-invariant influences – whether measured or unmeasured (Usami et al., 2019) – within-person relationships can still be confounded by external variables that change over time (Mund et al., 2021).

Assuming that time-varying factors exist and significantly influence the development of psychopathology, then the more pressing question becomes what these factors represent, and how such effects may emerge and change throughout development (Kan et al., 2019). Addressing this question from a dynamic mutualism perspective may argue that these effects are due to mutualistic processes that were not accounted for in our current models. For instance, numerous studies have documented links between poor executive functioning (EF) and psychopathology (Castellanos-Ryan et al., 2016; M. M. Martel et al., 2017; Moore et al., 2020; Wade et al., 2019), with a recent study suggesting that the development of poor EF and general psychopathology may be adequately described by a mutualism model, such that the effects of low EF compound throughout development and increase risk for developing multiple mental disorders in the future (Romer & Pizzagalli, 2021). Instead, and generally consistent with a common cause view of *p*, these effects could imply that after accounting for the more stable, communal features that *p* is often purported to reflect, other time-varying factors may still influence the expression of internalizing and externalizing symptoms to some degree. These time-varying effects could encompass a wide range of factors, extending from peer groups to parenting (Wichstrøm et al., 2017), to developmental genetic changes, that, if present, may impact within-person parameters (Mund et al., 2021; Pingault et al., 2015).

These considerations once again underscore the notion that more than one developmental process likely shapes the trajectories of psychopathology, with different developmental periods characterized by varying mechanisms or processes (e.g., McElroy, Belsky, et al., 2018; Wade et al., 2019). For instance, causal mechanisms in childhood that are distinct or embedded within a specific latent vulnerability (e.g., internalizing) may confer greater risk for developing broader expressions of psychopathology in adolescence by activating a small range of symptoms that slowly expand via interactions with each other, and/or through interactions with other genetic or environmental factors (Lahey et al., 2021). In adulthood, these symptom dynamics may be characterized by an entirely different process such as differentiation, in which symptoms become increasingly narrow with age (Lahey et al., 2004; Lilienfeld et al., 1994). Despite the simplistic nature of this scenario, it provides one example of how aspects of mutualism, differentiation, and elements of common cause positions can be combined to better understand or investigate possible accounts of development.

The attention to possible causal agents and the subsequent interplay between local-level symptoms also aligns with other recent frameworks for studying psychopathology, such as multi-causal (Kendler, 2019), or hybrid modeling approaches (e.g., Borsboom et al., 2019; Bringmann & Eronen, 2018; Fried & Cramer, 2017; Koss & Gunnar, 2018). Although definitions of hybrid models vary to some extent, Fried and Cramer (2017) proposed a hybrid modeling framework that integrates traditional network and latent variable techniques to unveil how common causes and direct symptom interactions may cooperatively influence the onset and maintenance of a disorder, respectively. Otherwise stated, causal factors (represented as latent variables) are theorized to initiate the expression of certain symptoms, which, in turn, may facilitate the development of new symptoms via local interactions (Fried & Cramer, 2017).

For example, the death of a relative (i.e., a hypothesized causal variable) may lead to symptoms of depression in some individuals (e.g., loss of appetite, sleep disturbances), that subsequently enables the disordered state to be maintained over time by promoting the development of other symptoms (e.g., sleep deprivation leads to increased anxiety; Pires et al., 2016). Future research may thus benefit from examining the extent to which different developmental frameworks can account for more specific elements of psychopathology, as well as how these proposed mechanisms may change throughout the lifespan.

Notwithstanding the promise that hybrid models may hold for future research, it is worth noting that the success of these models critically relies on the ability of researchers to identify and measure potential causal mechanisms, which has proven to be challenging with respect to *p* (van Bork et al., 2017). However, in cases where this may be feasible, residual network models (RNMs) provide one statistical solution for examining assumptions of hybrid models, as RNMs allow the network structure to be estimated after accounting for the influence that a latent variable has on its item covariances (Epskamp et al., 2017). Though more research in this area is needed, a recent study examining childhood maltreatment and eating disorder symptoms also proposed a hybrid modeling approach that integrates network analysis and mediation models, highlighting other viable solutions (Monteleone et al., 2019).

### Strengths and Limitations

The racially diverse sample and longitudinal study design represent key strengths that enabled the development of *p* to be probed in participants often underrepresented in research and during a key transitional period not previously examined. Likewise, although prior dynamic mutualism studies used large community samples, they relied on parental or teacher reports and only one study was considered adequately diverse with respect to nationality, ethnicity, and socioeconomic status. Thus, the diversity of the current sample and use of self-report data provided the opportunity to compare how these developmental processes may have differed based on sample characteristics and type of informant report.

Another key strength of the present study was the integration of different statistical techniques, including a model that disaggregated between- and within-person processes (Hamaker et al., 2015). While this differs from most *p*-factor studies that have used between-person techniques to study change, methods that attend to both intra-and interindividual trends are likely to yield more accurate descriptions of developing symptoms. Further, the combination of latent variable and network approaches may be especially fruitful for advancing research in this area, as the integration of techniques is more likely to describe the complexities of psychopathology than either approach alone (Eaton, 2015).

Despite these strengths, our study is subject to the following limitations. First, although our multi-method approach allowed for a more suitable test of mutualism theory compared to earlier studies, these analyses reflect a simplified version of the mutualism model of intelligence, and some assumptions of the theory (e.g., multiplier effects that are routed through the environment) were not directly tested. Likewise, given that mutualistic processes are not necessarily expected to increase during all points of development (Kievit, 2020), it is possible that our study failed to capture key dynamics between internalizing and externalizing that emerged during earlier stages of development. Second, our analyses were unable to incorporate other key forms of psychopathology (e.g., psychosis, personality disorders) that if included, may have led to different conclusions. In order to fully appreciate the development of co-occurring psychopathology, it will be critical for research to delineate changes in *p* when a more diverse array of symptoms are represented (Levin-Aspenson et al., 2021; Shields et al., 2020). Third, due to the nature of our sample, the generalizability of these results may be limited, and replications in samples that are diverse with respect to sex and gender are encouraged. In the same vein, future replications in clinical or high-risk samples will be equally beneficial, as rates of psychopathology in the current sample are expected to be lower compared to clinical populations.

Fourth, our index of substance use was based on the average frequency of alcohol, marijuana, and tobacco use and did not consider the quantity of use. Therefore, results pertaining to substance use may be less generalizable and are unlikely to distinguish between more normative or experimental use of alcohol or other drugs as opposed to problematic substance use (Deas et al., 2000). Fifth, findings were based on self-reported measures of psychopathology, which may lead to inflated correlations due to common method variance (Richardson et al., 2009) or overlapping diagnostic criteria (e.g., trouble concentrating; Milberger et al., 1995). Sixth, our internalizing factor was comprised of only two indicators and would be considered under-identified if separated from the larger model. Relatedly, because this factor was constructed based on symptoms of generalized anxiety and depression, our representation of internalizing may not be comparable to studies that have included both distress and fear disorders (e.g., Gomez et al., 2019; Krueger & Markon, 2006). Sixth, network models were estimated cross-sectionally and cannot speak to whether bidirectional feedback loops or self-reinforcing edges were present in the network structure. Consequently, data with sufficient timepoints for estimating multilevel networks will be beneficial for discerning directionality and examining whether feedback loops are present between internalizing and externalizing indices (Epskamp, Waldorp, et al., 2018).

## Conclusions

The *p*-factor is often conceptualized as an overarching predisposition to psychopathology, with on-going debate regarding a preferred statistical model for its study (Lahey et al., 2021). In exploring alternative theories of *p* and its development, the present study offers preliminary support for the use of a dynamic mutualism model in understanding the development of *p* and the internalizing-externalizing factors. In doing so, we hope to promote a more open dialogue surrounding the utility and substantive meaning of the *p*-factor, as well as encourage researchers to consider alternative frameworks and methodologies when investigating its development. Exploring different theoretical models may not only foster an increased understanding of the developmental mechanisms underlying *p*, but in turn may lead to novel insights into the prevention and treatment of psychopathology.

## Supplementary Material

1

## Figures and Tables

**Table 1.** Descriptive statistics and reliability

	ADHD	CD/ASPD	ODD	Substance Use	GAD	MDD
Age 14						
Mean	13.82	1.29	5.48	0.32	2.07	7.57
*SD*	7.99	2.10)	4.06	0.86	1.51	4.88
ω	.90	.77	.87	.74	.78	.77
Age 15						
Mean	13.71	1.42	5.50	0.50	2.13	7.26
*SD*	8.10	2.12	4.04	1.08	1.60	4.86
ω	.91	.76	.87	.78	.80	.79
Age 16						
Mean	12.95	1.29	5.05	0.66	2.10	6.89
*SD*	7.97	2.07	3.87	1.22	1.60	4.84
ω	.91	.77	.87	.72	.81	.80
Age 17						
Mean	12.16	1.17	4.74	0.86	2.07	6.49
*SD*	8.13	1.94	3.91	1.37	1.66	4.93
ω	.91	.68	.87	.68	.82	.83
Age 18						
Mean	12.21	1.36	4.91	1.23	1.70	6.01
*SD*	7.99	1.73	3.93	1.54	1.19	4.44
ω	.91	.75	.88	.64	.81	.86
Age 19						
Mean	12.02	1.22	4.52	1.43	1.75	6.31
*SD*	7.90	1.66	3.77	1.60	1.21	4.60
ω	.91	.75	.88	.61	.81	.85
Age 20						
Mean	12.34	1.18	4.63	1.61	1.82	6.70
*SD*	8.18	1.69	3.88	1.64	1.25	4.73
ω	.92	.75	.88	.57	.82	.86
Age 21						
Mean	12.31	1.10	4.55	1.78	1.84	6.83
*SD*	7.96	1.51	3.68	1.61	1.25	4.64
ω	.91	.71	.87	.56	.82	.85

*Note*. ADHD = attention-deficit hyperactivity disorder; CD/ASPD = conduct disorder/antisocial personality disorder traits; GAD = generalized anxiety disorder; MDD = major depressive disorder; ODD = oppositional defiant disorder; Substance Use = average frequency of alcohol, marijuana, and tobacco use; *SD* = standard deviation; ω = total omega reliability coefficient.

**Table 2.** Factor strength, reliability, and replicability based on confirmatory bifactor models at each age

Age	Factor	ECV	ω_H_/ω_HS_	Relative ω	ω/ω_S_	*H*
14	*P-*Factor	.63	.69	.82	.84	.84
	Internalizing	.52	.32	.54	.59	.37
	Externalizing	.31	.18	.21	.84	.66
15	*P-*Factor	.59	.66	.78	.84	.83
	Internalizing	.55	.35	.58	.61	.40
	Externalizing	.36	.21	.25	.84	.73
16	*P-*Factor	.61	.66	.79	.83	.82
	Internalizing	.53	.32	.54	.60	.37
	Externalizing	.34	.21	.26	.83	.62
17	*P-*Factor	.65	.68	.81	.84	.84
	Internalizing	.42	.27	.42	.63	.33
	Externalizing	.33	.21	.26	.82	.62
18	*P-*Factor	.69	.72	.82	.88	.90
	Internalizing	.34	.27	.33	.80	.39
	Externalizing	.29	.22	.27	.82	.50
19	*P-*Factor	.71	.73	.83	.88	.89
	Internalizing	.33	.26	.32	.81	.39
	Externalizing	.27	.21	.26	.82	.47
20	*P-*Factor	.72	.75	.84	.89	.89
	Internalizing	.31	.25	.31	.82	.37
	Externalizing	.27	.20	.24	.83	.47
21	*P-*Factor	.74	.76	.86	.88	.88
	Internalizing	.26	.21	.26	.82	.31
	Externalizing	.26	.19	.23	.82	.45

*Note*. ECV = Explained Common Variance; ω_H/_ω_HS_ = Omega Hierarchical and Subscale Omega Hierarchical; ω/ω_S_ = Omega and Omega Specific; Relative ω = relative Omega; *H* = construct replicability.

**Table 3.** Autoregressive and cross-lagged paths for the longitudinal bifactor model

	Est (SE)	β	Est (SE)	β	Est (SE)	β
*Age 14 → 15*	*P-factor*		*Internalizing*		*Externalizing*	
P-factor	1.05 (0.06)	.72***	0.22 (0.12)	.13	0.22 (0.13)	.16
Internalizing	0.14 (0.06)	.10*	1.30 (0.17)	.76***	−0.20 (0.06)	−.14**
Externalizing	0.16 (0.09)	.11	−0.39 (0.09)	−.23***	0.98 (0.15)	.68***
*Age 15 → 16*	*P-factor*		*Internalizing*		*Externalizing*	
P-factor	0.79 (0.06)	.72***	0.13 (0.11)	.09	0.05 (0.11)	.05
Internalizing	0.12 (0.05)	.12*	1.07 (0.19)	.86***	−0.17 (0.05)	−.19***
Externalizing	0.18 (0.10)	.16	0.01 (0.09)	.004	0.66 (0.12)	.65***
*Age 16 → 17*	*P-factor*		*Internalizing*		*Externalizing*	
P-factor	0.55 (0.06)	.63***	0.06 (0.10)	.04	0.54 (0.09)	.51***
Internalizing	0.14 (0.06)	.21*	0.88 (0.21)	.86***	−0.22 (0.09)	−.27*
Externalizing	−0.02 (0.07)	−.02	−0.11 (0.08)	−.08	0.56 (0.09)	.48***
*Age 17 → 18*	*P-factor*		*Internalizing*		*Externalizing*	
P-factor	0.38 (0.08)	.38***	0.35 (0.23)	.15	0.38 (0.12)	.21**
Internalizing	0.29 (0.09)	.45***	1.34 (0.83)	.88	−0.21 (0.13)	−.18
Externalizing	0.22 (0.07)	.26**	−0.13 (0.23)	−.07	1.22 (0.26)	.80***
*Age 18 → 19*	*P-factor*		*Internalizing*		*Externalizing*	
P-factor	0.30 (0.11)	.29**	0.01 (0.09)	.01	0.31 (0.09)	.23***
Internalizing	0.20 (0.09)	.46*	0.37 (0.18)	.76*	−0.03 (0.06)	−.05
Externalizing	0.24 (0.08)	.42**	−0.02 (0.07)	−.03	0.56 (0.11)	.76***
*Age 19 → 20*	*P-factor*		*Internalizing*		*Externalizing*	
P-factor	0.44 (0.10)	.44***	0.21 (0.13)	.21	0.24 (0.13)	.16
Internalizing	0.30 (0.10)	.33**	0.61 (0.13)	.65***	−0.33 (0.14)	−.24*
Externalizing	0.19 (0.10)	.24	−0.02 (0.12)	−.03	0.92 (0.20)	.78***
*Age 20 → 21*	*P-factor*		*Internalizing*		*Externalizing*	
P-factor	0.51 (0.12)	.46***	0.41 (0.16)	.38**	0.31 (0.15)	.20*
Internalizing	0.44 (0.16)	.42**	0.53 (0.19)	.51**	−0.20 (0.20)	−.14
Externalizing	0.13 (0.10)	.19	−0.14 (0.09)	−.20	0.79 (0.21)	.80***

Note.

* *p* < .05;

** *p* < .01;

*** *p* < .001.

**Table 4.** Goodness of fit and model comparisons for the random-intercept cross-lagged panel models (RI-CLPMs)

Model	SB-χ^2^ (YB)	*df*	R-CFI	R-TLI	R-RMSEA	AIC
1. Baseline (INT ≠ EXT)	2684.91*** (1.28)	887	.96	.95	.033	323698
2. Unidirectional (INT → EXT)	2648.73*** (1.28)	880	.96	.95	.033	323655
3. Unidirectional (INT → EXT)	2653.65*** (1.28)	880	.96	.95	.033	323672
4. Mutualism (INT ↔ EXT)	2630.18*** (1.28)	873	.96	.95	.033	32364

Model comparisons	Δχ^2^	Δ*df*	ΔR-CFI	ΔR-RMSEA
1 versus 2	32.14***	7	0	0
1 versus 3	31.34***	7	0	0
1 versus 4	52.97***	14	0	0
2 versus 4	18.45*	7	0	0
3 versus 4	22.88**	7	0	0

*Note*. INT = internalizing factor; EXT = externalizing factor; *df* = degrees of freedom; SB-χ^2^ = Satorra-Bentler corrected chi-square statistic; YB = Yuan-Bentler correction; R-CFI = robust comparative fit index; R-TLI = Robust Tucker-Lewis index; R-RMSEA = robust root-mean-square error of approximation; AIC = Akaike Information Criterion; Δχ^2^ = change in chi-square based on non-robust chi-square statistic.

* *p* < .05;

** *p* < .01;

*** *p* < .001.

**Table 5.** Parameter estimates for the bidirectional random-intercept cross-lagged panel model (RI-CLPM)

	Age 15		Age 16		Age 17		Age 18		Age 19		Age 20		Age 21	
	Est (*SE*)	β	Est (*SE*)	β	Est (*SE*)	β	Est (*SE*)	β	Est (*SE*)	β	Est (*SE*)	β	Est (*SE*)	β
*Autoregressions*														
INT → INT	0.48 (0.11)	.56***	0.53 (0.10)	.58***	0.46 (0.12)	.43***	0.66 (0.15)	.58***	0.30 (0.13)	.29*	0.43 (0.14)	.40**	0.50 (0.21)	.54*
EXT → EXT	0.52 (0.06)	.57***	0.55 (0.06)	.60***	0.40 (0.06)	.43***	0.19 (0.10)	.20*	0.23 (0.11)	.25*	0.16 (0.15)	.15	−0.18 (0.25)	−.17
*Cross-lags*														
INT → EXT	−0.01 (0.05)	−.02	−0.09 (0.07)	−.10	−0.03 (0.08)	−.03	0.06 (0.08)	.07	0.03 (0.07)	.04	0.18 (0.09)	.27*	0.48 (0.17)	.73**
EXT → INT	−0.21 (0.08)	−.23**	−0.14 (0.06)	−.16*	−0.10 (0.08)	−.09	−0.37 (0.16)	−.28*	0.06 (0.17)	.04	0.13 (0.22)	.07	0.10 (0.31)	.07

Correlations	Est (*SE*)	*r*
Between-person	5.67 (0.29)	.78***
Age 14	4.56 (0.38)	.60***
Age 15	2.77 (0.29)	.63***
Age 16	2.28 (0.26)	.65***
Age 17	2.28 (0.29)	.80***
Age 18	3.75 (0.28)	.82***
Age 19	3.75 (0.25)	.80***
Age 20	4.15 (0.31)	.85***
Age 21	3.43 (0.33)	.93***

*Note*. Est = unstandardized beta; SE = standard error; β = standardized beta; INT = Internalizing; EXT = Externalizing.

* *p* < .05;

** *p* < .01;

*** *p* < .001.

## Footnotes

1 For the purposes of this paper, co-occurrence rather than comorbidity is used as a more general term to describe the concurrent manifestation of two or more mental disorders (see Lilienfeld et al., 1994).

2 Importantly, although Lahey et al. (2015) previously constructed a bifactor model to examine the longitudinal associations of *p* in the same population-based sample of girls, their study focused on a younger developmental period (ages 5–11) and utilized parental reports rather than self-report data. Therefore, the present study not only extends results of Lahey et al. (2015) but builds upon prior findings that have examined the development of the *p*-factor in younger ages with teacher or parent reports (McElroy, Belsky, et al., 2018; Murray et al., 2016).

3 Of note, while positive, reciprocal interactions are predicted by dynamic mutualism, the presence of some negative or sparse interactions can still be consistent with mutualism and lead to a positive manifold in the data if a sufficient number of positive associations are present (van der Maas et al., 2017).

4 In the event that temporal associations were found to be driven by *p* predicting the specific factors, this was interpreted as evidence against mutualism.

5 Compared to the latent variable models, the reduced computational complexity in estimating the network models allowed for the average frequency of substance use to be separated into past year frequency of alcohol, marijuana, and tobacco use. Doing so enabled the associations across specific substances, as well as their relations with other internalizing and externalizing nodes, to be directly assessed in the network models.

6 The chi-bar-square test was used over the chi-square difference test, as this is a more appropriate test of differences in model fit when constraints are imposed on the bound of a given parameter (Stoel et al., 2006).
