# Supplementary material for: The general psychopathology factor (p) from adolescence to adulthood: Exploring the developmental trajectories of p using a multi-method approach
Source: Dev Psychopathol. Author manuscript; Available in PMC 2024 Apr 1. (PMC9832177; doi:10.1017/S0954579422000463)
Supplement: 1 [file NIHMS1800592-supplement-1.pdf]

SUPPLEMENTAL MATERIALS

Exploratory Models

Exploratory bifactor models were estimated at each age using the Psych package in R-Studio (Revelle & Revelle, 2015). Factors were extracted based on an orthogonal target rotation, whereby the rotation is influenced by prior theoretical assumptions about the factor structure (Marsh, Morin, Parker, & Kaur, 2014; Morin, Myers, & Lee, 2020). Specification of the target matrix was based on the internalizing and externalizing factor structure used in the confirmatory models, such that all items were targeted to load on the *p*-factor in addition to one of the specific factors. For example, internalizing items were set to have a zero-target loading on the externalizing factor and vice versa, which enabled cross-loadings to be freely estimated while still attempting to match the target matrix as closely as possible (Reise, Moore, & Haviland, 2010).

Exploratory Model Results

Standardized factor loadings are reported below in Table S1 by age. Overall, factor loadings on *p* increased over time for most constructs apart from ODD and frequency of substance use. Cross-loadings for internalizing and externalizing constructs were usually small and fell below .20 at all ages. ECV for the *p*-factor was similar between age 14 and age 21 and was characterized by greater fluctuations and less growth over time relative to the confirmatory models (Table S2). Similar to Murray and colleagues (2016), ECV and ω_{HS} for the internalizing and externalizing factors was substantially smaller compared to the *p*-factor. These estimates were also weaker relative to the confirmatory models. Construct replicability (*H*) for the *p*-factor was high. In contrast, *H* for internalizing and externalizing usually fell below recommended cut-offs of ≥.70 apart from externalizing at age 18 (H = .95).

Table S1. Standardized factor loadings for exploratory bifactor models by age.

Age	14	15	16	17	18	19	20	21
Factor 1 – Extracted General P-Factor								
GAD	.27	.28	.33	.42	.55	.55	.62	.64
MDD	.54	.55	.54	.60	.72	.71	.76	.77
ADHD	.71	.69	.75	.76	.88	.99	.89	.87
CD/ASPD	.59	.49	.52	.46	.62	.58	.60	.56
ODD	.96	.94	.85	.85	.76	.68	.72	.75
Substance Use	.30	.22	.24	.17	.20	.28	.23	.26
Factor 2 – Extracted Internalizing Specific Factor								
GAD	.47	.43	.49	.39	.53	.44	.35	.77
MDD	.52	.59	.49	.49	.46	.58	.58	.24
ADHD	.15	.13	.06	.11	.12	-.05	.03	.03
CD/ASPD	-.04	-.01	-.06	-.04	-.12	-.03	-.05	.01
ODD	-.08	-.08	-.02	-.09	-.04	.10	0	-.03
Substance Use	-.03	-.02	.01	.04	.03	-.02	.04	-.04
Factor 3 – Extracted Externalizing Specific Factor								
GAD	-.12	-.13	-.15	-.15	-.04	-.06	-.04	-.02
MDD	.06	.07	.09	.11	.03	.04	.03	.02
ADHD	.05	.09	.04	.11	.06	0	.10	.12
CD/ASPD	.52	.67	.58	.61	.30	.54	.47	.65
ODD	-.03	.10	.12	.22	.13	.24	.22	.16
Substance Use	.54	.61	.68	.54	.97	.47	.64	.40

Note. ADHD = attention-deficit/hyperactivity disorder; CD/ASPD = conduct disorder/antisocial personality disorder traits; GAD = generalized anxiety disorder; MDD = major depressive disorder; ODD = oppositional defiant disorder; Substance Use = average frequency of alcohol, marijuana, and tobacco use.

Table S2. Factor strength, reliability, and replicability based on exploratory bifactor models.

Age	Factor	ECV	ω_H/ω_{HS}	Relative ω	ω	*H*
14	P-Factor	.67	.71	.85	.84	.94
	Internalizing	.16	.06	.07		.41
	Externalizing	.18	.07	.08		.45
15	P-Factor	.59	.64	.77	.84	.90
	Internalizing	.16	.07	.08		.44
	Externalizing	.25	.13	.15		.59
16	P-Factor	.60	.66	.79	.83	.83
	Internalizing	.15	.06	.07		.39
	Externalizing	.25	.12	.14		.58
17	P-Factor	.64	.65	.79	.83	.83
	Internalizing	.13	.05	.06		.34
	Externalizing	.23	.13	.15		.53
18	P-Factor	.62	.74	.82	.90	.87
	Internalizing	.12	.05	.06		.41
	Externalizing	.26	.11	.13		.95
19	P-Factor	.71	.75	.85	.89	.99
	Internalizing	.14	.05	.06		.43
	Externalizing	.15	.08	.09		.43
20	P-Factor	.70	.74	.83	.89	.88
	Internalizing	.12	.05	.05		.40
	Externalizing	.18	.10	.11		.50
21	P-Factor	.68	.76	.85	.90	.88
	Internalizing	.16	.05	.05		.60
	Externalizing	.15	.09	.10		.49

Note. ECV = Explained Common Variance; ω_H/ω_{HS} = Omega Hierarchical and Subscale Omega Hierarchical; ω = Omega total for the *p*-factor; Relative ω = relative omega; *H* = construct replicability.

Longitudinal Measurement Invariance

Configural, metric (i.e., weak; equivalence of factor loadings), and scalar (i.e., strong; equivalence of factor loadings and intercepts) levels of invariance were evaluated for the superior fitting bifactor and random-intercept cross-lagged panel model (RI-CLPM). Specifically, we constructed a series of increasingly restrictive models that were sequentially compared with chi-square differences tests and changes in alternative fit indices (Chen, 2007). Due to our larger sample size, support for longitudinal invariance was not based on the significance of the chi-square difference test (Meade, Johnson, & Braddy, 2008). Instead, invariance was supported based on recommended changes in CFI and RMSEA fit statistics that were ≤ 0.01 and 0.015, respectively (Chen, 2007; Cheung & Rensvold, 2002). As the current study was most concerned with making inferences on structural relationships rather than latent means, establishment of partial metric invariance was considered sufficient.

For the configural models, the factor loadings, intercepts, and residual variances were free to vary, and models were identified by fixing factor means to 0 and variances to 1. Metric invariance was assessed by constraining factor loadings to be equal to their respective constructs across ages 14-21. In the event that metric invariance was not supported, we attempted to establish partial invariance by releasing constraints on factor loadings with the largest modification index. If partial metric invariance was supported, we next tested assumptions of scalar invariance by imposing constraints on item intercepts and comparing this model to the partial metric invariance model. Given the changes in item content from the CD items (measured from ages 14–17) to the ASPD items (replaced CD items starting at age 18), as well as anxiety items that were assessed by the SCARED (measured from ages 14-17) and ASRI-4 (measured

from ages 18-21), two separate constraints were applied. Specifically, CD/ASPD traits and anxiety indicators were constrained, respectively, between ages 14-17 and ages 18-21.

Measurement Invariance Results

Tests of measurement invariance for the bifactor with autoregressive and cross-lagged paths and bidirectional RI-CLPM can be found in Table S3. For the bifactor model, constraining factor loadings to be equal across ages 14-21 resulted in unacceptable changes in model fit based on changes in CFI but not RMSEA (ΔCFI = .015; ΔRMSEA = .008). Consequently, we inspected modification indices, which indicated that CD/ASPD traits and ODD should be freely estimated on the *p*-factor over time. Doing so resulted in acceptable changes in CFI and RMSEA (ΔCFI = .008; ΔRMSEA = .004), providing support for partial metric invariance.

Scalar invariance was examined by imposing constraints across factor loadings in addition to item intercepts. Constraining item intercepts to be equal led across age led to significant decrements in model fit based on changes in CFI and RMSEA (ΔCFI = .030; ΔRMSEA = .013), and thus modification indices were inspected to guide which intercepts should be released. Modification indices were largest for CD/ASPD traits and frequency of substance use, and these intercepts were freed to test whether partial scalar invariance was supported. Freely estimating these intercepts led to acceptable changes in model fit (ΔCFI = .030; ΔRMSEA = .013), yielding support for partial scalar invariance.

With respect to the RI-CLPM, constraining factor loadings to be equal led to negligible decreases in model fit (ΔCFI = .003; ΔRMSEA = .001), thereby supporting metric invariance. However, imposing constraints across item intercepts resulted in substantially poorer fit to the data (ΔCFI = .024; ΔRMSEA = .000), and modification indices suggested that CD/ASPD traits and substance use frequency should be released. Akin to the bifactor model, releasing item

intercepts for these two constructs resulted in acceptable changes in alternative fit indices (ΔCFI = .001; ΔRMSEA = .000).

In sum, measurement non-invariance was more pronounced for the bifactor model compared to the RI-CLPM, though appeared to be small overall. Violations of invariance were primarily attributable to changes in CD/ASPD traits, average frequency of substance use, and to a lesser extent, ODD. These findings are consistent with other adolescent studies that have reported measurement non-invariance for drug and tobacco use loadings on the *p*-factor (Castellanos-Ryan et al., 2016).

In examining the average use across specific substances, mean-levels of alcohol, marijuana, and tobacco use all increased over time; however, frequency of alcohol and tobacco use experienced greater mean-level changes throughout development relative to marijuana use. Although mean-level changes were still relatively small, average increases in the frequency of tobacco and alcohol use were particularly notable between ages 17 and 18 and ages 20 and 21, respectively. These increases may reflect change in accessibility, as girls were able to purchase tobacco products starting at age 18 and alcohol at age 21.

Table S3. Measurement invariance for the best fitting bifactor model and RI-CLPM.

	SB- χ^2 (YB)	*df*	$\Delta\chi^2$	Δdf	R-CFI	R-RMSEA	ΔCFI	ΔRMSEA
Bifactor Model (autoregressive and cross-lagged paths)								
Configural	1633.08 (1.29)	801	--	--	.984	.023	--	--
Metric	2409.17 (1.28)	881	611.19^{***}	80	.969	.031	.015	.008
Metric partial	2067.32 (1.28)	868	351.83^{***}	67	.976	.027	.008	.004
Scalar	3568.05 (1.26)	908	1918.40^{***}	40	.946	.040	.030	.013
Scalar partial	2373.64 (1.27)	895	370.51^{***}	27	.970	.030	.006	.003
RI-CLPM (bidirectional)								
Configural	2630.18 (1.28)	873	--	--	.964	.033	--	--
Metric	2817.90 (1.28)	899	177.59^{***}	26	.961	.034	.003	.001
Scalar	4007.58 (1.28)	925	1494.50^{***}	26	.937	.043	.024	.009
Scalar partial	2881.99 (1.28)	912	70.71^{***}	13	.960	.034	.001	0

Note. SB- χ^2 = Satorra-Bentler corrected chi-square statistic; YB = Yuan Bentler correction; *df* = degrees of freedom R-CFI = robust comparative fit index; R-RMSEA = robust root-mean-square error of approximation; Δdf = change in degrees of freedom; $\Delta\chi^2$ = change in chi-square based on non-robust chi-square statistic. *p < .05, **p < .01, ***p < .001.

Table S4. Estimated factor loadings and standard errors for bifactor models.

	Model 1: Autoregressive paths only		Model 2: Autoregressive and cross-lagged paths	
	Est (*SE*)	β	Est (*SE*)	β
Age 14				
P-Factor				
GAD	0.50 (0.04)***	.33	0.50 (0.05)***	.33
MDD	2.96 (0.11)***	.60	2.95 (0.12)***	.60
ADHD	6.34 (0.19)***	.79	6.03 (0.20)***	.76
CD/ASPD	0.06 (0.00)***	.45	0.08 (0.01)***	.53
ODD	3.18 (0.09)***	.80	3.26 (0.10)***	.81
Substance Use	0.13 (0.03)***	.16	0.20 (0.03)***	.23
Internalizing				
GAD	0.56 (1.73)	.37	0.88 (0.07)***	.58
MDD	2.61 (8.20)	.53	1.74 (0.15)***	.35
Externalizing				
ADHD	0.63 (0.29)*	.08	0.66 (0.38)	.08
CD/ASPD	0.11 (0.01)***	.82	0.09 (0.01)***	.62
ODD	0.82 (0.16)***	.20	0.71 (0.19)***	.18
Substance Use	0.38 (0.05)***	.45	0.46 (0.05)***	.54
Age 15				
P-Factor				
GAD	0.31 (0.02)***	.30	0.25 (0.04)***	.23
MDD	1.85 (0.08)***	.58	1.78 (0.11)***	.53
ADHD	4.20 (0.16)***	.79	4.16 (0.17)***	.76
CD/ASPD	0.04 (0.00)***	.42	0.05 (0.01)***	.49
ODD	2.06 (0.08)***	.79	2.19 (0.09)***	.80
Substance Use	0.11 (0.02)***	.15	0.15 (0.04)***	.20
Internalizing				
GAD	0.38 (1.19)	.40	0.57 (0.05)***	.61
MDD	1.63 (5.03)	.56	1.05 (0.12)***	.37
Externalizing				
ADHD	0.31 (0.16)	.06	-0.17 (0.31)	-.03
CD/ASPD	0.08 (0.01)***	.90	0.06 (0.01)***	.58
ODD	0.49 (0.09)***	.19	0.21 (0.15)	.07
Substance Use	0.34 (0.04)***	.48	0.42 (0.05)***	.56
Age 16				
P-Factor				

GAD	0.31 (0.02)***	.32	0.21 (0.04)***	.21
MDD	1.63 (0.08)***	.56	1.47 (0.10)***	.49
ADHD	3.69 (0.16)***	.77	3.63 (0.20)***	.74
CD/ASPD	0.03 (0.00)***	.42	0.05 (0.01)***	.54
ODD	1.79 (0.08)***	.78	1.92 (0.09)***	.80
Substance Use	0.11 (0.02)***	.15	0.22 (0.05)***	.29
Internalizing				
GAD	0.27 (0.84)	.38	0.44 (0.05)***	.44
MDD	1.12 (3.43)	.51	0.83 (0.11)***	.28
Externalizing				
ADHD	0.26 (0.16)	.05	-0.42 (0.33)	-.08
CD/ASPD	0.07 (0.01)***	.86	0.05 (0.01)***	.53
ODD	0.42 (0.08)***	.18	0.02 (0.16)	.01
Substance Use	0.37 (0.05)***	.49	0.44 (0.05)***	.52
Age 17				
P-Factor				
GAD	0.37 (0.02)***	.35	0.35 (0.04)***	.29
MDD	1.93 (0.08)***	.61	1.99 (0.13)***	.57
ADHD	4.39 (0.16)***	.83	4.47 (0.17)***	.78
CD/ASPD	0.04 (0.00)***	.46	0.04 (0.00)***	.41
ODD	1.91 (0.07)***	.76	1.99 (0.08)***	.73
Substance Use	0.14 (0.02)***	.16	0.14 (0.04)***	.14
Internalizing				
GAD	0.28 (0.86)	.33	0.34 (0.06)***	.44
MDD	1.32 (3.98)	.53	0.64 (0.14)***	.31
Externalizing				
ADHD	0.14 (0.15)	.03	0.17 (0.31)	.04
CD/ASPD	0.06 (0.01)***	.70	0.04 (0.00)***	.48
ODD	0.43 (0.07)***	.18	0.39 (0.14)**	.17
Substance Use	0.40 (0.04)***	.48	0.44 (0.04)***	.54
Age 18				
P-Factor				
GAD	0.43 (0.02)***	.53	0.35 (0.05)***	.41
MDD	2.22 (0.09)***	.73	1.97 (0.17)***	.63
ADHD	5.11 (0.17)***	.92	4.72 (0.32)***	.86
CD/ASPD	0.14 (0.01)***	.57	0.12 (0.01)***	.50
ODD	2.00 (0.07)***	.73	1.83 (0.11)***	.67
Substance Use	0.22 (0.02)***	.20	0.21 (0.04)***	.19
Internalizing				
GAD	0.20 (0.62)	.38	0.16 (0.10)	.44

MDD	1.14 (3.48)	.57	0.42 (0.27)	.31
Externalizing				
ADHD	-0.01 (0.09)	0	0.10 (0.24)	.03
CD/ASPD	0.08 (0.01)***	.52	0.06 (0.01)***	.45
ODD	0.34 (0.07)***	.19	0.33 (0.11)**	.22
Substance Use	0.33 (0.05)***	.49	0.32 (0.05)***	.53
Age 19				
P-Factor				
GAD	0.44 (0.02)***	.54	0.36 (0.04)***	.43
MDD	2.29 (0.08)***	.73	1.97 (0.18)***	.63
ADHD	5.01 (0.14)***	.92	4.65 (0.22)***	.89
CD/ASPD	0.13 (0.01)***	.58	0.11 (0.01)***	.47
ODD	1.86 (0.06)***	.72	1.64 (0.09)***	.65
Substance Use	0.20 (0.02)***	.19	0.12 (0.05)*	.11
Internalizing				
GAD	0.31 (0.95)	.40	0.37 (0.06)***	.49
MDD	1.70 (5.19)	.57	1.13 (0.19)***	.39
Externalizing				
ADHD	0.13 (0.12)	.03	0.02 (0.39)	.01
CD/ASPD	0.09 (0.01)***	.49	0.07 (0.01)***	.42
ODD	0.43 (0.06)***	.21	0.40 (0.13)**	.20
Substance Use	0.42 (0.05)***	.50	0.47 (0.04)***	.56
Age 20				
P-Factor				
GAD	0.46 (0.02)***	.58	0.41(0.05)***	.48
MDD	2.28 (0.09)***	.75	2.13(0.18)***	.65
ODD	1.81 (0.07)***	.71	1.73(0.11)***	.65
ADHD	4.92 (0.18)***	.92	4.82(0.32)***	.86
Substance Use	0.19 (0.02)***	.18	0.17(0.04)***	.15
CD/ASPD	0.13 (0.01)***	.58	0.11(0.01)***	.49
Internalizing				
GAD	0.29 (0.89)	.39	0.39 (0.09)***	.46
MDD	1.54 (4.71)	.55	1.12 (0.29)***	.36
Externalizing				
ODD	0.39 (0.07)***	.21	0.41 (0.15)**	.24
ADHD	0.09 (0.13)	.02	0.22 (0.51)	.06
Substance Use	0.39 (0.05)***	.52	0.39 (0.07)***	.54
CD/ASPD	0.08 (0.01)***	.51	0.07 (0.01)***	.45
Age 21				
P-Factor				

GAD	0.46 (0.02)***	.62	0.37 (0.05)***	.47
MDD	2.12 (0.09)***	.75	2.03 (0.26)***	.69
ADHD	4.48 (0.19)***	.92	4.15 (0.36)***	.84
CD/ASPD	0.11 (0.01)***	.61	0.09 (0.01)***	.49
ODD	1.70 (0.09)***	.74	1.57 (0.13)***	.67
Substance Use	0.20 (0.02)***	.21	0.14 (0.05)**	.14
Internalizing				
GAD	0.24 (0.73)	.39	0.40 (0.09)***	.49
MDD	1.17 (3.59)	.50	0.80 (0.19)***	.27
Externalizing				
ADHD	0.05 (0.12)	.01	0.33 (0.27)	.09
CD/ASPD	0.06 (0.01)***	.46	0.06 (0.01)***	.45
ODD	0.32 (0.09)***	.20	0.41 (0.13)**	.24
Substance Use	0.32 (0.07)***	.47	0.37 (0.07)***	.52

Note. ADHD = attention-deficit hyperactivity disorder; CD/ASPD = conduct disorder/antisocial personality disorder; GAD = generalized anxiety disorder; MDD = major depressive disorder; ODD = oppositional defiant disorder; Substance Use = average frequency of alcohol, marijuana, and tobacco use.

**p* < .05, ***p* < .01, ****p* < .001.

Table S5. Centrality correlations across ages 14, 17, and 20 for between-person networks.

Variable	1	2	3	4	5	6	7	8
1. B_{14}	1							
2. C_{14}	.87** [.43, .98]	1						
3. EI_{14}	.67 [-.06, .93]	.87** [.43, .98]	1					
4. B_{17}	.87** [.41, .98]	.88** [.47, .98]	.55 [-.25, .90]	1				
5. C_{17}	.76* [.13, .95]	.95** [.73, .99]	.77* [.13, .95]	.87** [.43, .98]	1			
6. EI_{17}	.58 [-.21, .91]	.82* [.26, .97]	.94** [.70, .99]	.49 [-.32, .89]	.81* [.24, .96]	1		
7. B_{20}	.24 [-.56, .81]	.42 [-.40, .87]	.33 [-.49, .84]	.36 [-.46, .85]	.56 [-.24, .91]	.53 [-.27, .90]	1	
8. C_{20}	.47 [-.35, .88]	.69 [-.04, .94]	.55 [-.25, .90]	.58 [-.21, .91]	.82* [.27, .97]	.72* [.03, .94]	.90** [.55, .98]	1
9. EI_{20}	.35 [-.47, .85]	.48 [-.34, .88]	.47 [-.35, .88]	.31 [-.50, .83]	.63 [-.13, .93]	.72* [.04, .95]	.84** [.35, .97]	.88** [.45, .98]

Note. B = Betweenness; C = Closeness; EI = Expected Influence; Subscripts reflect the age in which the network was estimated.

Values in square brackets are the 95% confidence interval for each correlation. *$p < .05$, **$p < .01$, ***$p < .001$.

Table S6. Centrality correlations across ages 14, 17, and 20 for within-person networks.

Variable	1	2	3	4	5	6	7	8
1. B_{14}	1							
2. C_{14}	.79* [.18, .96]	1						
3. EI_{14}	.76* [.13, .95]	.91** [.57, .98]	1					
4. B_{17}	.18 [-.60, .78]	.56 [-.24, .91]	.65 [-.09, .93]	1				
5. C_{17}	.52 [-.29, .90]	.87** [.41, .98]	.89** [.48, .98]	.85** [.35, .97]	1			
6. EI_{17}	.24 [-.56, .81]	.62 [-.14, .92]	.62 [-.16, .92]	.72* [.02, .94]	.75* [.10, .95]	1		
7. B_{20}	.93** [.63, .99]	.74* [.07, .95]	.76* [.13, .95]	.05 [-.68, .73]	.49 [-.33, .89]	.19 [-.60, .79]	1	
8. C_{20}	.76* [.12, .95]	.92** [.62, .99]	.91** [.58, .98]	.52 [-.29, .90]	.89** [.48, .98]	.60 [-.19, .92]	.80* [.22, .96]	1
9. EI_{20}	.68 [-.06, .94]	.79* [.19, .96]	.78* [.17, .96]	.38 [-.45, .85]	.76* [.11, .95]	.69 [-.02, .94]	.76* [.11, .95]	.91** [.57, .98]

Note. B = Betweenness; C = Closeness; EI = Expected Influence; Subscripts reflect the age in which the network was estimated.

Values in square brackets are the 95% confidence interval for each correlation. *p < .05, **p < .01, ***p < .001.

Table S7. Estimated edges and edge weight difference tests based on NetworkComparisonTests.

	**Between-Person Networks**								**Within-Person Networks**							
	GAD	MDD	ADHD	CD/ASPD	ODD	FAU	FMU	FTU	GAD	MDD	ADHD	CD/ASPD	ODD	FAU	FMU	FTU
Age 14 – bolded values reflect edges that were significantly different between ages 14 and 17																
GAD	--								--							
MDD	.31	--							.24	--						
ADHD	.09	.18	--						.11	.18	--					
CD/ASPD	0	0	.09	--					0	0	**.23**	--				
ODD	0	.21	.49	.35	--				0	.24	.43	.14	--			
FAU	0	.11	0	.17	0	--			0	0	0	**.26**	0	--		
FMU	-.08	0	0	.25	0	.21	--		0	0	-.07	**.18**	0	.26	--	
FTU	0	0	0	0	0	.26	.41	--	0	0	0	**.09**	0	**0**	**.35**	--
Age 17 – bolded values reflect edges that were significantly different between ages 17 and 20																
GAD	--								--							
MDD	**.32**	--							**.17**	--						
ADHD	.16	**.23**	--						.16	**.22**	--					
CD/ASPD	-.10	.09	**.10**	--					**0**	0	.10	--				
ODD	0	.18	**.47**	.32	--				0	.17	.41	.17	--			
FAU	0	0	0	.14	0	--			0	0	0	.13	0	--		
FMU	0	0	0	.20	0	.27	--		0	0	0	0	.08	.26	--	
FTU	0	0	0	.09	0	**.22**	.29	--	0	0	0	0	0	.17	.15	--
Age 20 – bolded values reflect edges that were significantly different between ages 14 and 20																
GAD	--								--							
MDD	**.47**	--							.30	--						
ADHD	.14	**.36**	--						.09	**.34**	--					
CD/ASPD	0	0	**.28**	--					**.09**	0	.21	--				
ODD	0	.15	**.35**	**.21**	--				0	.13	.36	.18	--			
FAU	0	**0**	0	.18	0	--			0	0	0	.14	0	--		
FMU	0	0	0	.16	.10	.21	--		0	0	0	.15	0	.24	--	
FTU	0	0	0	.12	0	**.09**	**.24**	--	0	0	0	0	0	.11	**.13**	--

Note. ADHD = attention deficit/hyperactivity disorder; CD/ASPD = conduct disorder/antisocial personality disorder; FAU = frequency of alcohol use; FMU = frequency of marijuana use; FTU = frequency of tobacco use; GAD = generalized anxiety disorder; MDD = major depressive disorder; ODD = oppositional defiant disorder.

Figure S1. Nonparametric bootstrapped confidence intervals (CIs) of the estimated edges by age and network type.

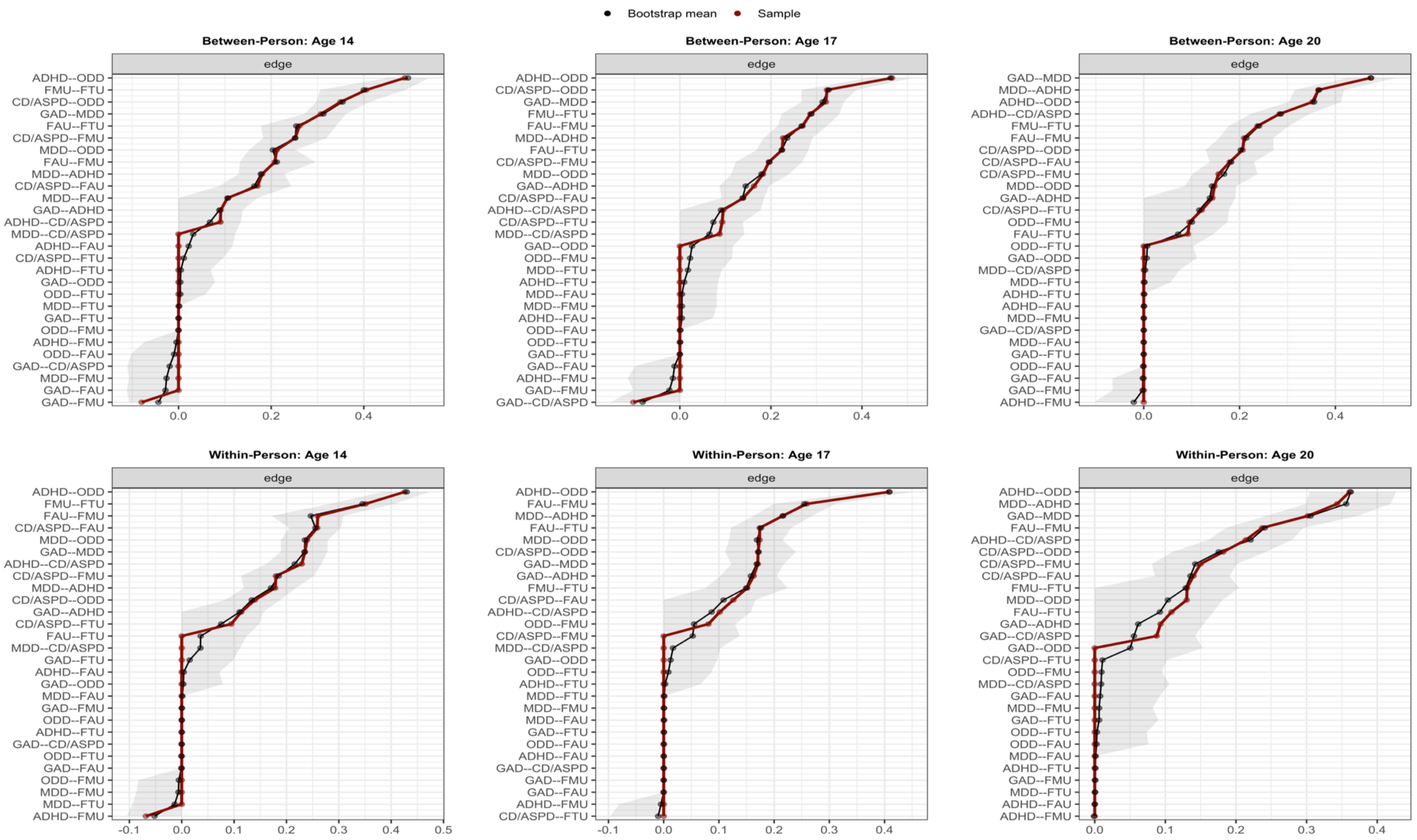

Note. The shaded area in gray represents the 95% bootstrapped CI. ADHD = attention deficit/hyperactivity disorder; CD/ASPD = conduct disorder/antisocial personality disorder; FAU = frequency of alcohol use; FMU = frequency of marijuana use; FTU = frequency of tobacco use; GAD = generalized anxiety disorder; MDD = major depressive disorder; ODD = oppositional defiant disorder.

Figure S2. Centrality stability of the estimated networks by age and type.

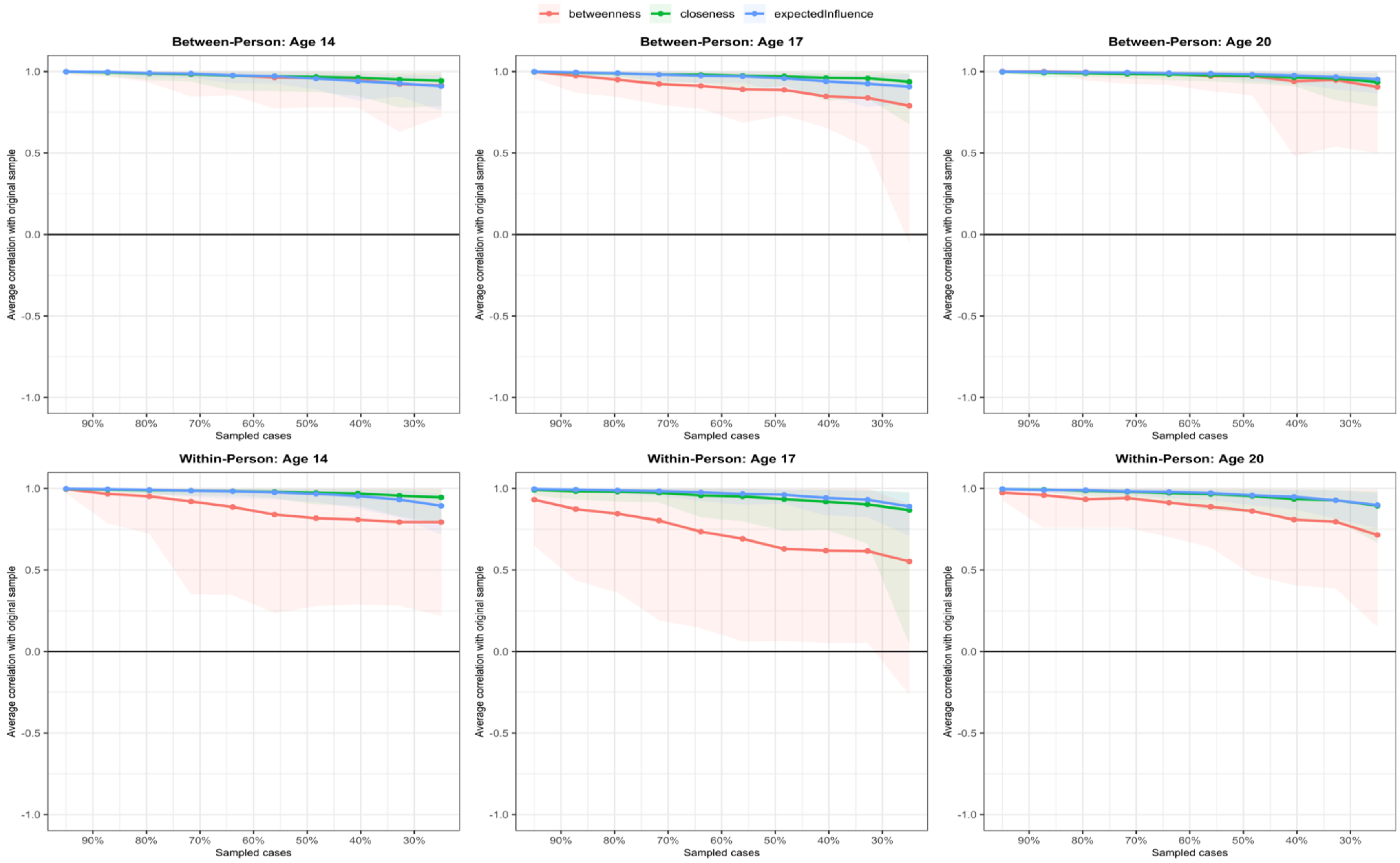

Note. Centrality stability reflects the average correlations between the original network sample and case-dropped sample. Plotted lines represent means and the shaded areas around the lines represent 95% confidence intervals (CIs).

Figure S3. Nonparametric bootstrapped difference test for edges by age and network type.

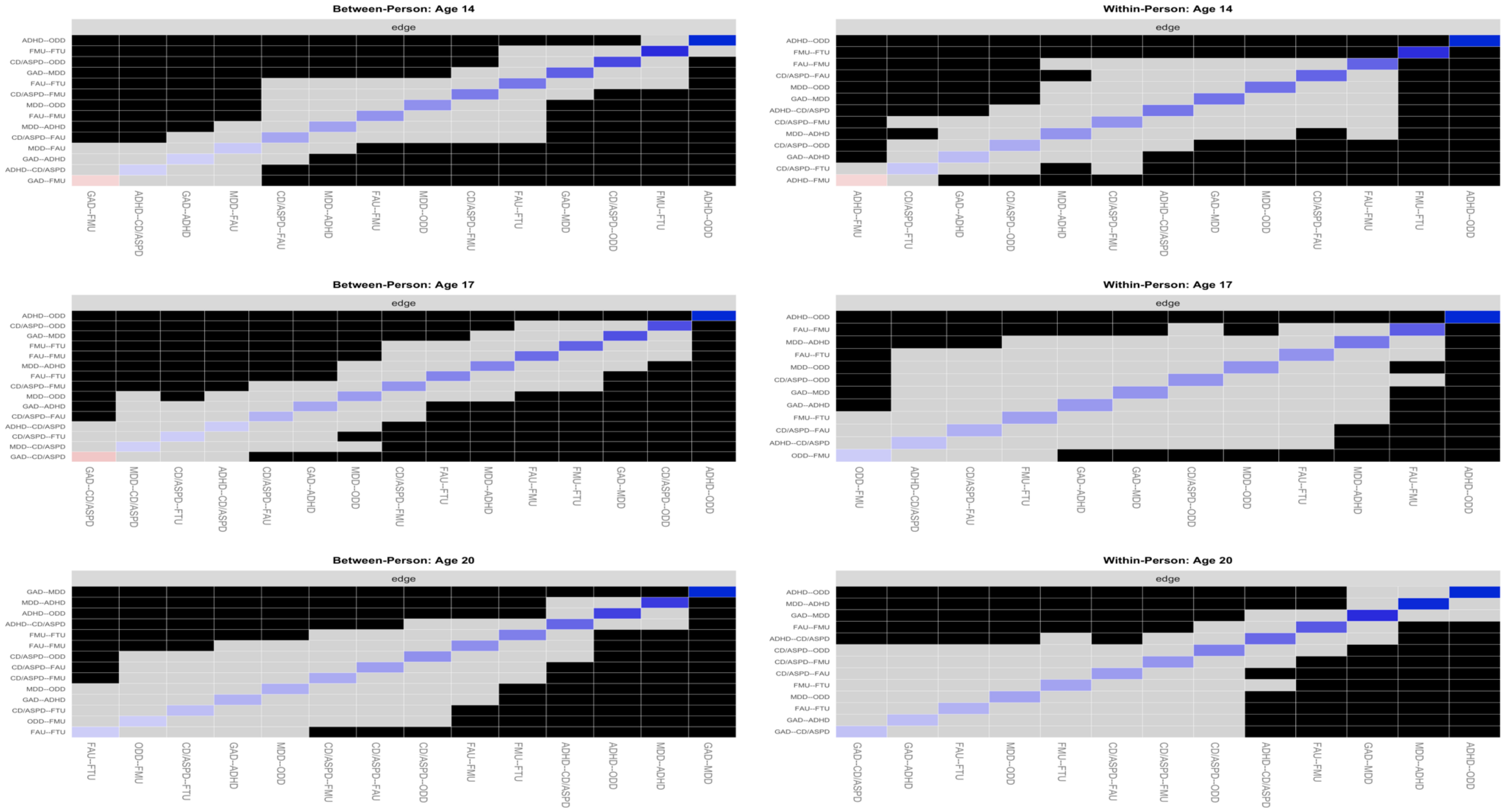

Note. Black shaded boxes indicate a statistically significant difference ($p < .05$). Darker color saturation on the diagonal reflects stronger edge associations. ADHD = attention deficit/hyperactivity disorder; CD/ASPD = conduct disorder/antisocial personality disorder; FAU = frequency of alcohol use; FMU = frequency of marijuana use; FTU = frequency of tobacco use; GAD = generalized anxiety disorder; MDD = major depressive disorder; ODD = oppositional defiant disorder.

Figure S4. Nonparametric bootstrapped difference test for betweenness by age and network type.

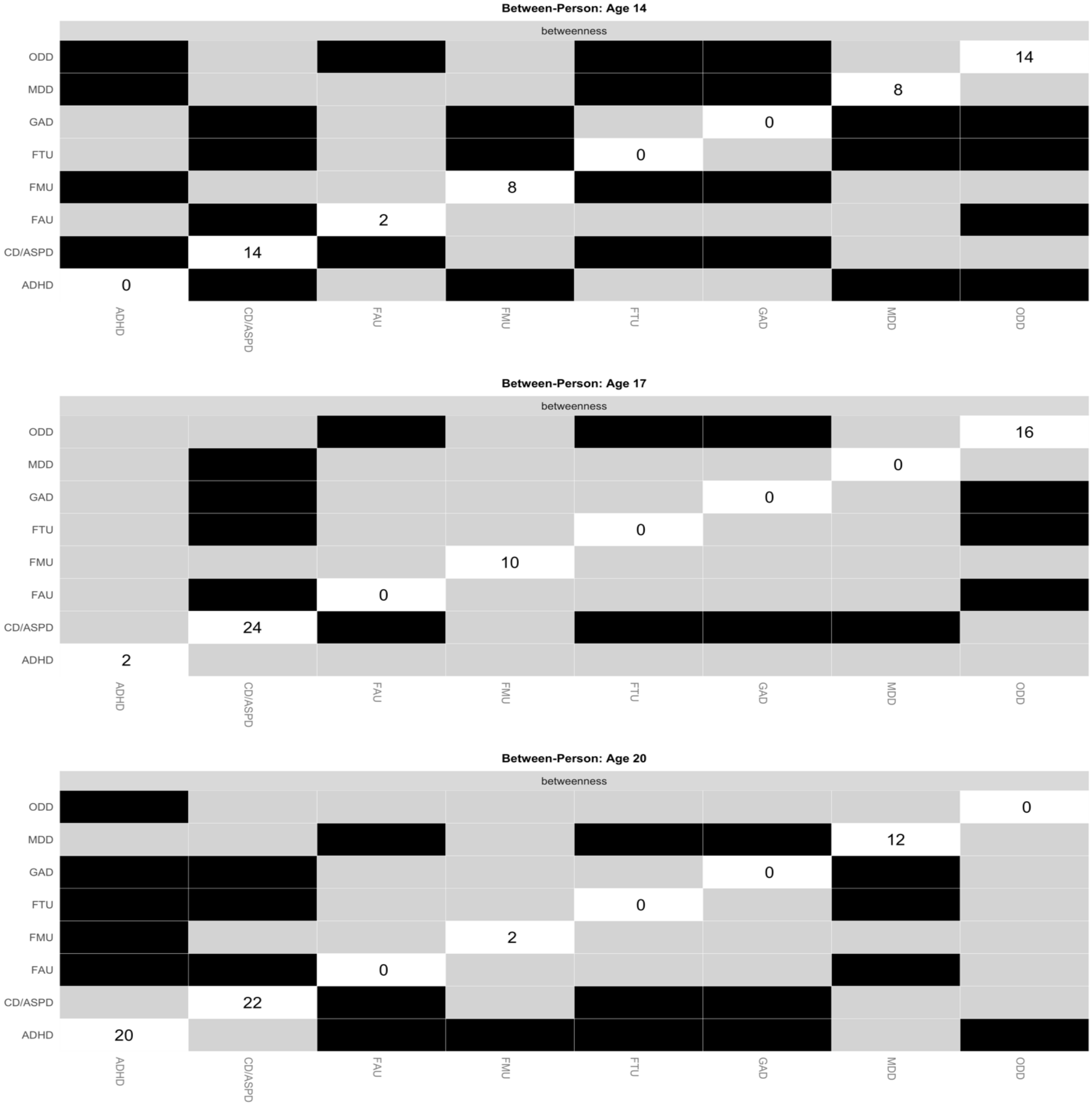

Note. The black shaded boxes indicate a statistically significant difference ($p < .05$). Within-person network estimates for betweenness were considered unstable and therefore were omitted. Values on the diagonal are the raw betweenness estimates for each node. ADHD = attention deficit/hyperactivity disorder; CD/ASPD = conduct disorder/antisocial personality disorder; FAU = frequency of alcohol use; FMU = frequency of marijuana use; FTU = frequency of tobacco use; GAD = generalized anxiety disorder; MDD = major depressive disorder; ODD = oppositional defiant disorder.

Figure S5. Nonparametric bootstrapped difference test for closeness by age and network type.

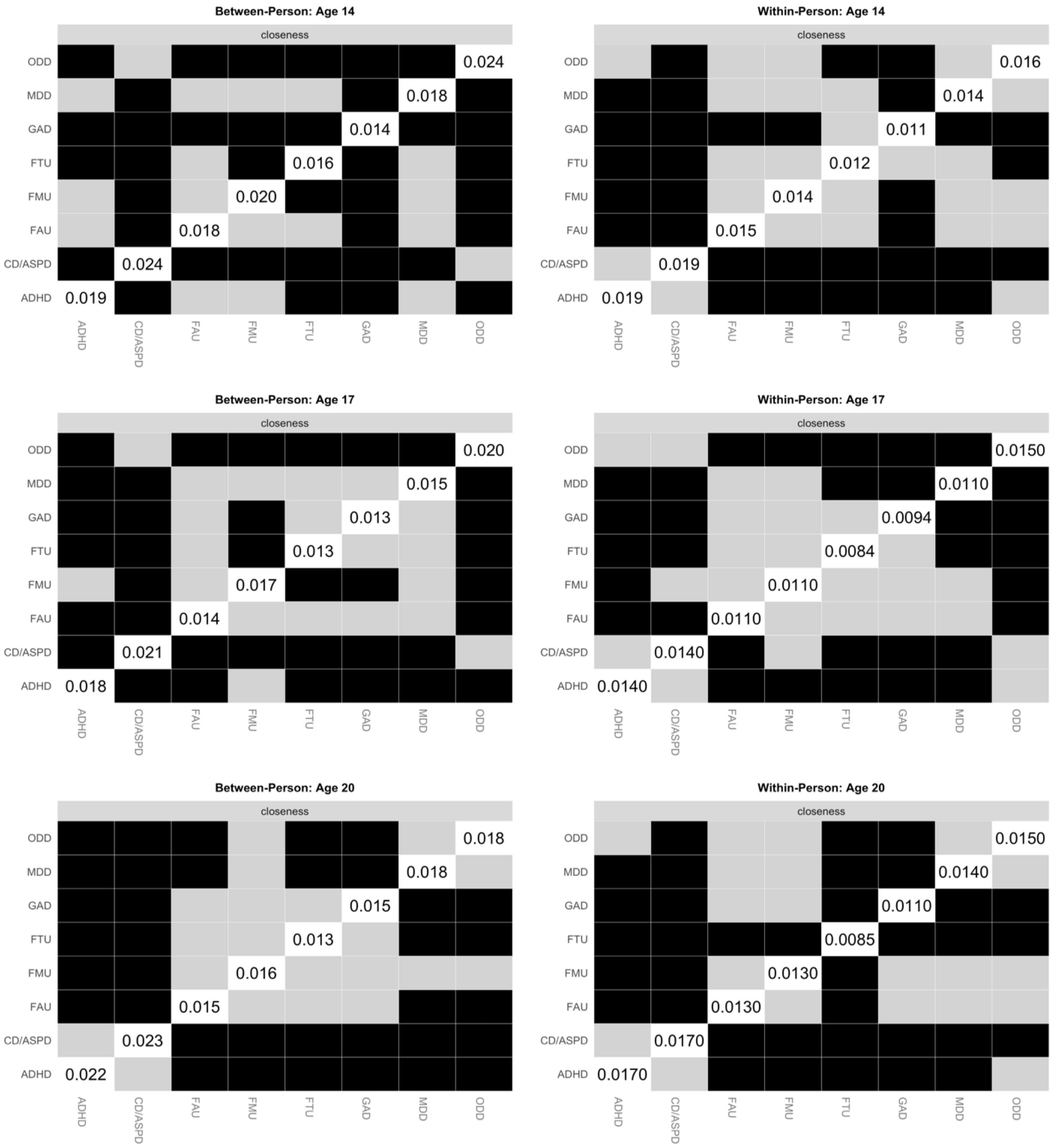

Note. The black shaded boxes indicate a statistically significant difference ($p < .05$). Values on the diagonal are the raw betweenness estimates for each node. ADHD = attention deficit/hyperactivity disorder; CD/ASPD = conduct disorder/antisocial personality disorder; FAU = frequency of alcohol use; FMU = frequency of marijuana use; FTU = frequency of tobacco use; GAD = generalized anxiety disorder; MDD = major depressive disorder; ODD = oppositional defiant disorder.

Figure S6. Nonparametric bootstrapped difference test for EI by age and network type.

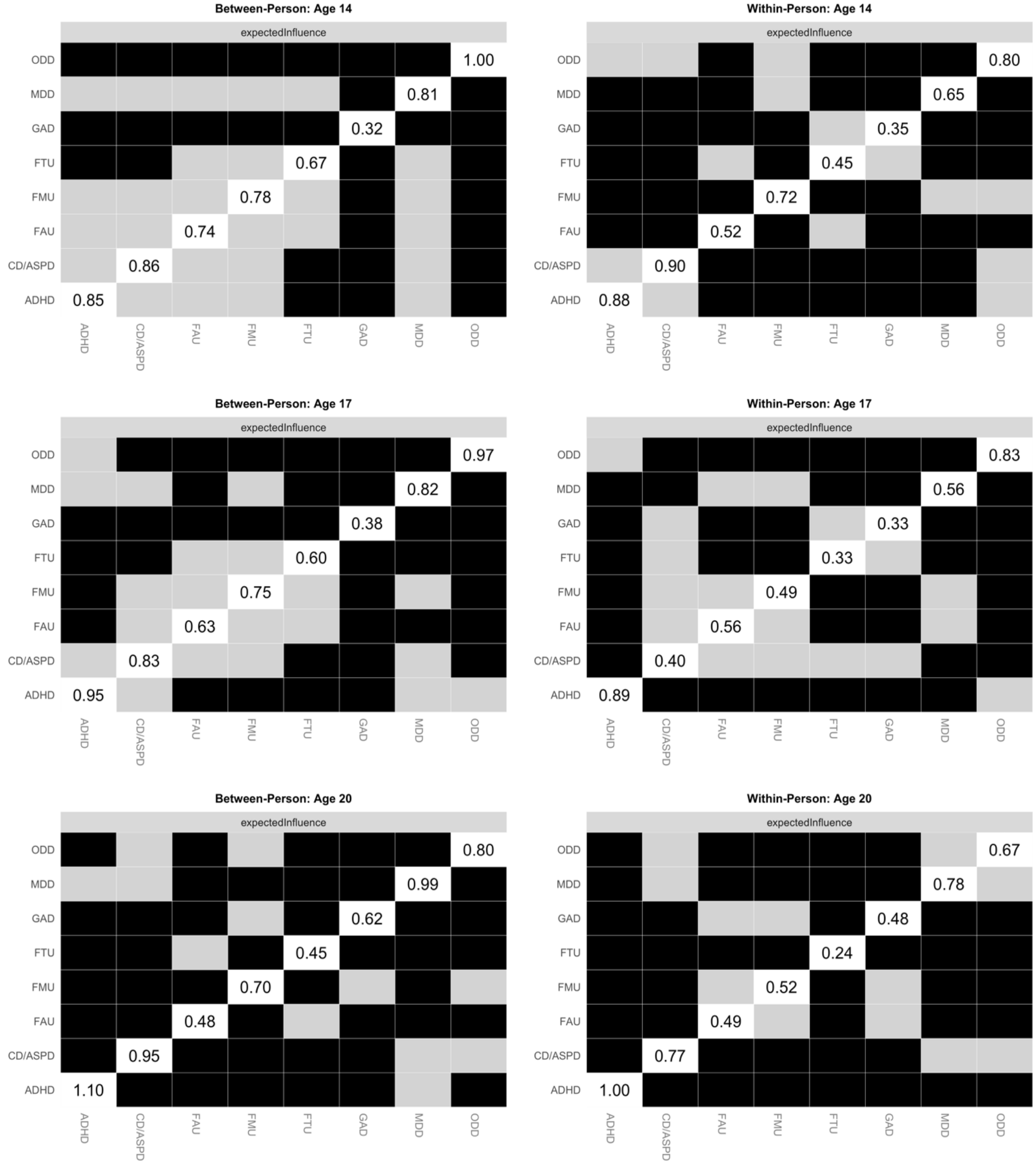

Note. The black shaded boxes indicate a statistically significant difference ($p < .05$). Values on the diagonal are the raw expectedInfluence (EI) estimates for each node. ADHD = attention deficit/hyperactivity disorder; CD/ASPD = conduct disorder/antisocial personality disorder; FAU = frequency of alcohol use; FMU = frequency of marijuana use; FTU = frequency of tobacco use; GAD = generalized anxiety disorder; MDD = major depressive disorder; ODD = oppositional defiant disorder.

References

Castellanos-Ryan, N., Brière, F. N., O'Leary-Barrett, M., Banaschewski, T., Bokde, A., Bromberg, U., . . . Gallinat, J. (2016). The structure of psychopathology in adolescence and its common personality and cognitive correlates. *Journal of abnormal psychology, 125*(8), 1039. doi:dx.doi.org/10.1037/abn0000193

Chen, F. F. (2007). Sensitivity of goodness of fit indexes to lack of measurement invariance. *Structural equation modeling: a multidisciplinary journal, 14*(3), 464-504. doi:doi.org/10.1080/10705510701301834

Cheung, G. W., & Rensvold, R. B. (2002). Evaluating goodness-of-fit indexes for testing measurement invariance. *Structural Equation Modeling, 9*(2), 233-255. doi:doi.org/10.1207/S15328007SEM0902_5

Marsh, H. W., Morin, A. J., Parker, P. D., & Kaur, G. (2014). Exploratory structural equation modeling: An integration of the best features of exploratory and confirmatory factor analysis. *Annual review of clinical psychology, 10*, 85-110. doi:doi.org/10.1146/annurev-clinpsy-032813-153700

Meade, A. W., Johnson, E. C., & Braddy, P. W. (2008). Power and sensitivity of alternative fit indices in tests of measurement invariance. *Journal of Applied Psychology, 93*(3), 568. doi:doi.org/10.1037/0021-9010.93.3.568

Morin, A. J., Myers, N. D., & Lee, S. (2020). Modern factor analytic techniques: Bifactor models, exploratory structural equation modeling (ESEM), and bifactor-ESEM. *Handbook of sport psychology*, 1044-1073. doi:doi.org/10.1002/9781119568124.ch51

Reise, S. P., Moore, T. M., & Haviland, M. G. (2010). Bifactor models and rotations: Exploring the extent to which multidimensional data yield univocal scale scores. *Journal of personality assessment, 92*(6), 544-559. doi:doi.org/10.1080/00223891.2010.496477

Revelle, W., & Revelle, M. W. (2015). Package 'psych'. *The comprehensive R archive network, 337*, 338.
